# Olive leaf extract effect on cardiometabolic profile among adults with prehypertension and hypertension: a systematic review and meta-analysis

**DOI:** 10.7717/peerj.11173

**Published:** 2021-04-07

**Authors:** Muhammad Asyraf Ismail, Mohd Noor Norhayati, Noraini Mohamad

**Affiliations:** 1 Department of Family Medicine, Health Campus, Universiti Sains Malaysia, Kubang Kerian, Kelantan, Malaysia; 2 Department of Family Medicine, School of Medical Sciences, Health Campus, Universiti Sains Malaysia, Kubang Kerian, Kelantan, Malaysia; 3 School of Dental Sciences, Health Campus, Universiti Sains Malaysia, Kubang Kerian, Kelantan, Malaysia

**Keywords:** Olive leaf extract, Olea europea, Olive leaves extracts, Phenolic compounds, Hypertension, Cardiometabolic profile, Blood pressure, Oleuropein, Polyphenols, Olive leaves

## Abstract

**Background:**

This systematic review and meta-analysis aimed to determine the effectiveness of olive leaf extract on cardiometabolic profiles among prehypertensive and hypertensive groups.

**Methodology:**

The Cochrane central register of controlled trials, Medline (1966 to April week 1, 2020), Embase (1966 to April week 1, 2020) and trial registries for relevant randomized clinical trials were used. Published and unpublished randomized clinical trials were reviewed and evaluated. Random effects models were used to estimate the continuous outcomes and mean differences (MDs); both with 95% confidence intervals (CIs). The primary outcomes were changes in systolic and diastolic BP. The secondary outcomes were changes in lipid profile, glucose metabolism, inflammatory markers for CVD, kidney and liver functions safety parameters. We assessed the data for risk of bias, heterogeneity, sensitivity, reporting bias and quality of evidence.

**Results:**

Five trials were included involving 325 patients aged 18–80 years. Two trials involved high-income countries and three trials involved moderate-income countries. The analysis performed was based on three comparisons. No significant changes were found between systolic or diastolic blood pressure (BP) for the first comparison, 1,000 mg per day for a combined formulation of olive leaf extract versus a placebo. The second comparison, 500 mg per day of olive leaf extract versus placebo or no treatment, showed a significant reduction in systolic BP over a period of at least 8 weeks of follow up (MD −5.78 mmHg, 95% CI [−10.27 to −1.30]) and no significant changes on diastolic BP. The third comparison, 1,000 mg per day of olive leaf extract versus placebo shows no significant difference but an almost similar reduction in systolic BP (−11.5 mmHg in olive leaf extract and −13.7 mmHg in placebo, MD 2.2 mmHg, 95% CI [−0.43–4.83]) and diastolic BP (−4.8 mmHg in olive leaf extract and −6.4 mmHg in placebo, MD 1.60 mmHg, 95% CI [−0.13–3.33]). For secondary outcomes, 1,000 mg per day of olive leaf extract versus captopril showed a reduction in LDL (MD −6.00 mg/dl, 95% CI [−11.5 to −0.50]). The 500 mg per day olive leaf extract versus placebo showed a reduction in inflammatory markers for CVD IL-6 (MD −6.83 ng/L, 95% CI [−13.15 to −0.51]), IL-8 (MD −8.24 ng/L, 95% CI [−16.00 to −0.48) and TNF-alpha (MD −7.40 ng/L, 95% CI [−13.23 to −1.57]).

**Conclusions:**

The results from this review suggest the reduction of systolic BP, LDL and inflammatory biomarkers, but it may not provide a robust conclusion regarding the effects of olive leaf extract on cardiometabolic profile due to the limited number of participants in the included trials.

**Review registrations:**

PROSPERO CDR 42020181212.

## Introduction

Cardiovascular disease (CVD) is the leading cause of death worldwide and is estimated at around 17.9 million lives in 2016, with heart attack and stroke constituting around 85% of the deaths (WHO, 2017). High blood pressure (BP) is the leading risk factor for CVD, chronic kidney disease, and diabetes in every international region, causing more than 40% of worldwide deaths from these diseases in 2010. It has been proven that cardiovascular risk rises in a strong, continuous and independent manner as BP levels increase, starting at 115/75 mmHg with suboptimal systolic BP (>115 mmHg) estimated to be responsible for 62% of cerebrovascular disease and 49% of coronary heart disease (Franco et al., 2005). Therefore, controlling BP will reduce cardiovascular risk by 20% to 25% for myocardial infarction, 35% to 40% for stroke and 50% for heart failure (Antonakoudis et al., 2007). In the local settings, the data from a national health morbidity survey (NHMS, 2015) showed that Malaysian adults at least 18 years old have a high prevalence of cardiovascular risk factors, including being overweight or obese, smoking, and having hypercholesterolemia, hypertension and diabetes mellitus (MOH, 2015).

The Mediterranean diet which rich in plant-based foods, fish and olive oil, had been shown to reduce cardiovascular risk factors, such as BP, lipid profiles, oxidative stress, endothelial dysfunction and antithrombotic profiles (Lopez-Miranda et al., 2010). Beyond olive oil derived from olive fruit, leaves of the olive tree (Olea Europaea) found mainly in the Mediterranean region also confers beneficial effects (Vogel et al., 2014). These leaves have a high content of phenolic compounds whereby the most abundant compound is oleuropein, followed by hydroxytyrosol, the flavone-7-glucosides of luteolin and apigenin, and verbascoside (Benavente-García et al., 2000). These phenolic compounds can be extracted after drying and milling via several techniques, including solid–liquid extraction by maceration and soxhlet extraction using water-methanol mixes or hexane to produce olive leaf extract (Lockyer et al., 2012). By comparison, olive leaves contain between 1–14% oleuropein compared to 0.005% to 0.12% in olive oil (Vogel et al., 2014). In addition, the phenolic compounds are 145 mg/100 g fresh leaf compared to 110 mg/100 g olive fruit and 23 mg/100 ml EVOO (Lockyer et al., 2017). Therefore, these leaves are more likely to provide beneficial effects, increasing human antioxidant capacity while acting as an antihypertensive, cardioprotective and anti-inflammatory that also reduces cholesterol (Vogel et al., 2014). Oleuropein provides cardioprotective effects via few mechanisms. It is said to have antioxidant and anti-inflammatory activity via inhibition of low-density lipoproteins oxidation and free radical scavenging at the cellular level by inhibiting the production of superoxide anions, a respiratory burst of neutrophils, platelet aggregation, production of thromboxane and leukotriene B4 by neutrophils (Omar, 2010). The anti-atherogenic activity of oleuropein acts through the reduced formation of short-chain aldehydes and reduced formation of malondialdehyde-lysine and 4-hydroxynonenal-lysine adducts, thus protect the apoprotein layer (Omar, 2010). Furthermore, oleuropein also protects atherosclerosis by reducing monocytoid cell adhesion to stimulated endothelium and vascular cell adhesion molecule-1 (VCAM-1) mRNA protein, which was essential in the early steps of atherogenesis (Omar, 2010). While there are no reports on the mechanism in human, in animal models it has been shown that oleuropein can protect the paraventricular nucleus (PVN) of the hypothalamus from oxidative stress by improving mitochondrial function through the activation of the Nrf2-mediated signaling pathway, thus, provide a promising strategy to treat hypertension (Sun, Frost & Liu, 2017). Furthermore, at the animal research level, olive leaf provides hypotensive activity via vasodilator effect (Zarzuelo et al., 1991). In addition, it reduces blood pressure by ameliorating the release of nitric oxide, antioxidant and sympatholytic activities (Nekooeian, Khalili & Khosravi, 2014).

As there were growing interests in olive leaf extract, the purpose of this review is to determine the effects of olive leaf extract as a supplement on the cardiometabolic profiles of prehypertensive and hypertensive patients. This meta-analysis’s primary outcomes were to evaluate the effect of olive leaf extract on systolic and diastolic blood pressure changes. Moreover, it would provide an additional supplement to treat hypertension other than conventional anti-hypertensive drugs. This meta-analysis’s secondary outcomes were to evaluate additional synergistic benefits of olive leaf extract on lipid profiles, glucose metabolism, and inflammatory markers for CVD. Other outcomes were to evaluate the kidney and liver function levels to indicate the safety of the olive leaf extract supplementation.

## Materials & Methods

We conducted this systematic review according to the protocol previously published in the PROSPERO register (http://www.crd.york.ac.uk/PROSPERO), [CDR 42020181212]. The types of studies included were randomized control trials (RCTs) comparing olive leaf extract with placebo, antihypertensive drugs or no treatment. We included blinded and open-label studies.

### Eligibility criteria

The inclusion criteria were adults aged 18 years old of any sex and any ethnicity considered eligible if diagnosed with elevated BP (≥ 120/80 mmHg; classified as prehypertension and hypertension based on the JNC-7). The types of interventions were olive leaf extract (either alone or in combination with other active ingredients), placebo, antihypertensive drugs or no treatment. The extract was administered orally, in either tablet or liquid form. The follow-up period for primary outcomes was at least six weeks after treatment. The exclusion criteria were patients with cardiovascular complications and stroke.

### Search strategy

We searched the Cochrane Central Register of Controlled Trials (CENTRAL; 2020, Issue 4) and MEDLINE (1966 to April week 1, 2020) (see appendix 1). We restricted the publications to those published in the English language only and searched the reference list of identified RCTs and review articles to find unpublished trials or trials not identified by electronic searches. We also searched for ongoing trials through the World Health Organization (WHO) International Clinical Trials Registry Platform (ICTRP; http://www.who.int/ictrp/en) and (NLM; http://www.clinicaltrials.gov).

### Trial selection

Two review authors (MAI, NMN) scanned the titles and abstracts from the searches and obtained full-text articles when they appeared to meet the eligibility criteria, or when there was insufficient information to assess eligibility. We assessed the eligibility of the trials independently and documented any reasons for exclusion. We resolved any disagreements between the review authors by discussion, contacting the authors for clarification if needed.

### Data extraction

From each of the selected trials, we extracted study settings, participant characteristics (age, sex, and ethnicity), methodology (the number of participants randomized and analyzed, the duration of follow-up), whether olive leaf extract was used alone or in combination with other active ingredients, and the method used for diagnosing prehypertension and hypertension. Predefined primary outcomes were changes in systolic BP and diastolic BP. The secondary outcomes were changes in lipid profiles (Total cholesterol (TC), Triglyceride (TG), LDL, and HDL), inflammatory markers for CVD (IL-6 and IL-8), TNF-alpha, kidney and liver function safety parameters (creatinine, aspartate aminotransferase (AST), alanine aminotransferase (ALT) and glucose metabolism (fasting blood sugar (FBS), homeostatic model of assessment-insulin resistance (HOMA-IR) and insulin).

### Risk of bias assessment

We assessed the risk of bias based on random sequence generation, allocation concealment, blinding of participants and personnel, blinding of outcome assessors, completeness of outcome data, the selectivity of outcome reporting and other bias (Higgins et al., 2019).

### Statistical analysis

We performed the meta-analysis using Review Manager 5.3 software (The Nordic Cochrane Centre, Copenhagen) and reported the results of the random-effects model. Since our meta-analysis measures continuous outcomes, we used the inverse variance for the outcomes. We drew forest plots for the trials with numerical outcomes using mean differences (MD) and 95% confidence intervals (CI) (Higgins et al., 2019). There was no missing data from the trials. We were able to retrieve data on one trial via a supplementary file attached to the article (Wong et al., 2014).

The planned subgroup analyses would have compared prehypertension with hypertension and high dose (1,000 mg per day) with a low dose (500 mg per day). However, we were unable to perform these analyses due to insufficient trials. We were also unable to perform a sensitivity analysis to investigate the impact of bias risk for sequence generation and allocation concealment of the included studies due to insufficient trials.

### Assessment of heterogeneity

We assessed the presence of heterogeneity in two steps. First, we assessed apparent heterogeneity at face value by comparing populations, settings, interventions and outcomes. Second, we assessed statistical heterogeneity through the *I*^2^ statistic (Higgins et al., 2019). We used the following guide to interpret heterogeneity, as mentioned in the Cochrane Handbook for Systematic Reviews of Interventions (Higgins et al., 2019) 0% to 40% signified negligible importance, 30% to 60% signified moderate heterogeneity, 50% to 90% indicated substantial heterogeneity and 75% to 100% indicated considerable heterogeneity.

### Grading evidence quality

We assessed evidence quality for primary and secondary outcomes according to GRADE methodology (Schünemann, Guyatt & Oxman, 2013) for risk of bias, inconsistency, indirectness, imprecision and publication bias. The quality of evidence was classified as one of four certainty levels: very low, low, moderate or high. Quality could be downgraded depending on the presence of four factors: (i) limitations in the design and implementation of available studies, (ii) evidence indirectness, (iii) unexplained result heterogeneity or inconsistency and (iv) imprecise results.

## Results

### Study descriptions

**Figure 1:**
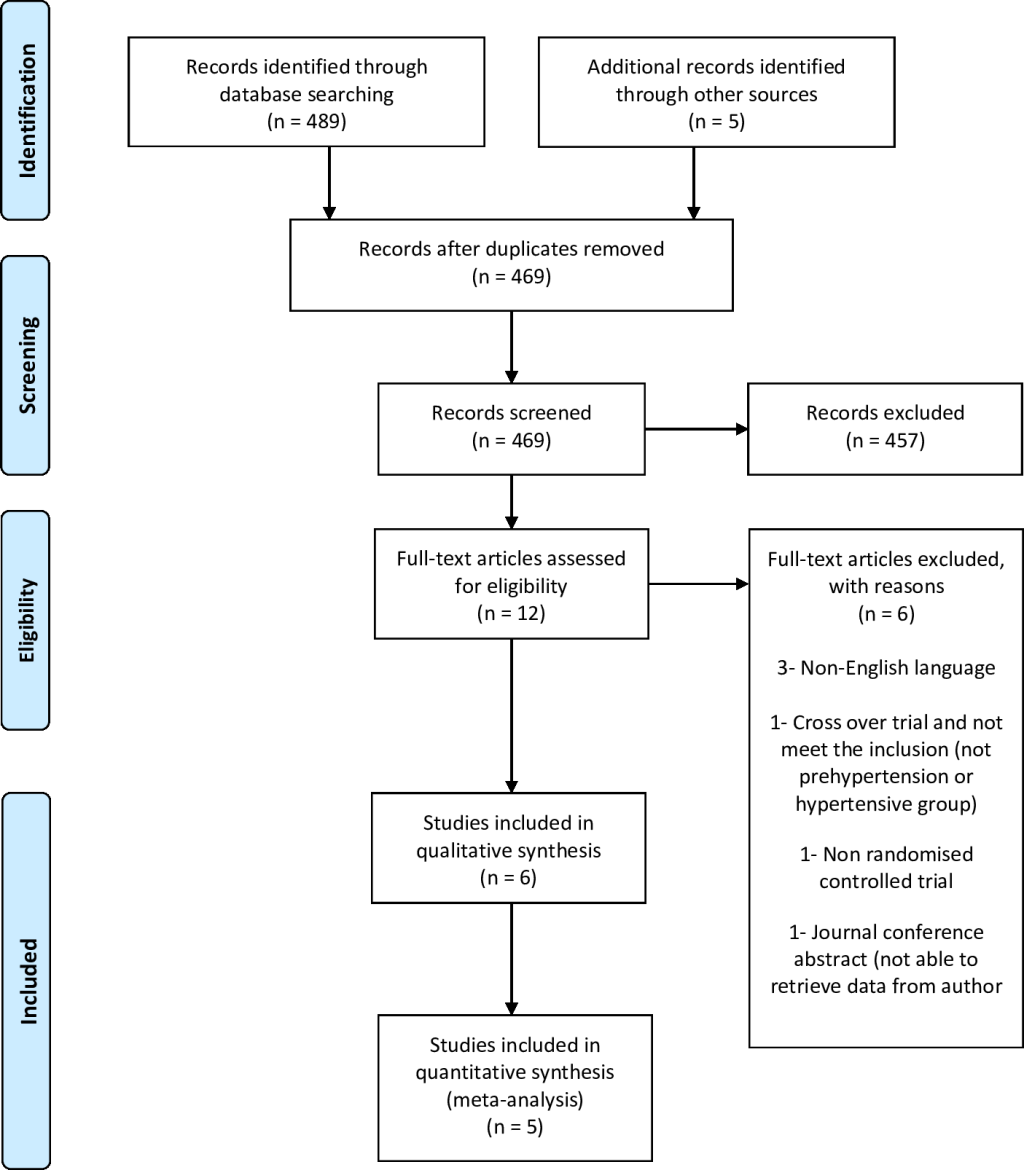
Flow of trials through study.

We retrieved 489 records from the electronic database search and five from other sources (Fig. 1) and screened a total of 494 records. We reviewed full copies of 12 studies, identifying six articles with the potential of meeting the review inclusion criteria and excluding the other six. Of those excluded, three were published in a non-English language (Cabrera-Vique et al., 2015; Saberi, Kazemisaleh & Bolurian, 2008; Schulz, 2011), one did not involve a prehypertensive or hypertensive group (De Bock et al., 2012), one was a non-RCT (Zam & Ali, 2017) and one was a conference abstract (Saibandith et al., 2016). We were not able to retrieve the data for the conference abstract after contacting the authors (Saibandith et al., 2016). A trial was also excluded due to being a crossover trial that we were unable to retrieve data on after contacting the author (Lockyer et al., 2017). Therefore, we only included five trials in this review.

### Included studies

The five trials included in this review (Javadi et al., 2019; Perrinjaquet-Moccetti et al., 2008; Susalit et al., 2011; Wong et al., 2014; Yaghoobzadeh et al., 2020) had met the eligibility criteria. One trial included monozygotic twins with untreated suboptimal blood pressure, exceeding 120 mmHg systolic or 80 mmHg diastolic at rest (Perrinjaquet-Moccetti et al., 2008). One trial included stage-1 hypertension either naïve or being under treatment with any anti-hypertensive medication and exclusions were participants with a history of secondary hypertension, presence of target-organ damage, second- or third-degree heart block, valvular heart disease, diabetic subjects, hepatic dysfunction, pregnant and breastfeeding female subjects (Susalit et al., 2011). One trial included participants with BP between 130–160 mmHg systolic and 85–100 mmHg diastolic and excluded participants that smoke or took nicotine therapy, taking anti-hypertensive medication or insulin, pregnant or currently breastfeeding and currently consuming dietary supplements containing extracts of olive leaf, green coffee bean or beet (Wong et al., 2014). One trial included participants with hypertension and exclusions were hypertensive patients who had complications such as diabetes, kidney disease, and hypo and/or hyperthyroidism (Javadi et al., 2019). One trial included patients with essential hypertension (Yaghoobzadeh et al., 2020). Two trials had declared funding from olive leaf extract manufacturers (Perrinjaquet-Moccetti et al., 2008; Susalit et al., 2011; Wong et al., 2014). Table 1 shows the characteristics of the included studies.

**Table 1:** Characteristics of included studies.

Study reference	Country	Study design	Age/ Mean age	Male/ Female (%)	Number of participants included	Number of participants completed the intervention	Participants in intervention group	Participants in comparator group	Indication	Drugs	Comparator	Duration of trials	Outcome
Javadi et al., 2019	Iran	Randomized, double-blind placebo controlled clinical trial	30–60 years old Olive leaf extract: 53.8 ± 8.0 Placebo: 55.6 ± 8.8	45/55	60	60	30	30	Hypertensive patients	Olive leaf extract 250 mg, one tablet twice daily	Placebo, one tablet twice daily	12 weeks	1. Metabolic parameters-FBS and Insulin, HOMA-IR 2. Biomarkers of inflammation-TNF-alpha, IL-6 and 1L-8 3. Liver function- AST, ALT 4. Kidney function- Creatinine
Yaghoobzadeh et al., 2020	Iran	Randomized, double-blind placebo-controlled clinical trial	30-60 years old Olive leaf extract: 52.9 ± 10.3 Placebo: 57.9 ± 10.8	50/50	60	60	30	30	Essential hypertension	Olive leaf extract 250 mg, one tablet twice per day	Placebo, one tablet twice per day	12 weeks	1. BP 2. Blood lipids 3. Oxidative stress (total antioxidant capacity (TAC), glutathione (GSH), Malondialdehyde (MDA))
Wong et al., 2014	Australia	Randomized, double-blind, placebo-controlled cross-over	18–80 years old 58.5 ± 10.7	54/46	39	37	19	18	Blood pressure of 130–160 mmHg systolic and 85–100 mmHg diastolic	Combined formulation of Olive leaf extract 500 mg, green coffee bean extract 100 mg and 150 mg of beetroot powder per tablet, two tablets daily	Placebo, two tablets daily	(Data taken only at 6 weeks before cross- over of treatment arm)	1. BP 2. Blood lipids 3. Fasting blood glucose, insulin levels and HOMA-IR
Susalit et al., 2011	Indonesia	Randomized, double-blind active-controlled clinical trial	20-65 years old Olive leaf extract: 51.5 ± 5.8 Captopril: 49.7 ± 6.8	16.7/83.3	179	148	72	76	stage 1 hypertension with systolic blood pressure of 140-159 mmHg and diastolic blood pressure of either <90 mmHg or in between 90 and 99 mmHg	Olive leaf extract 500 mg, one tablet twice daily	Captopril 12.5 mg–25 mg, one tablet twice daily	8 weeks	1. BP 2. Lipid profile 3. Safety endpoints-liver function, renal function, Adverse effects
Perrinjaquet-Moccetti et al., 2008	Germany	Open, controlled, parallel-group, co-twin study	18 and 60 years old Olive leaf extract: 35.7 ± 14.8 Control (lifestyle modification): 38.1 ± 14.7	30/70	20 Monozygotic twins	20 Monozygotic twins completed	10	10	Untreated suboptimal blood pressure ≥120 mmHg systolic or 80 mmHg diastolic	Olive leaf extract 500 mg versus lifestyle modification (no medication) once daily		8 weeks	1. BP 2. Blood lipids 3. body weight

### Participants

Two trials were conducted in high-income countries (Perrinjaquet-Moccetti et al., 2008; Wong et al., 2014) and three trials were conducted in moderate-income countries (Javadi et al., 2019; Susalit et al., 2011; Yaghoobzadeh et al., 2020). Three trials recruited patients from healthcare settings (Javadi et al., 2019; Susalit et al., 2011; Yaghoobzadeh et al., 2020) and two trials recruited patients from a research institute (Perrinjaquet-Moccetti et al., 2008; Wong et al., 2014). Two trials reported the exclusion of participants due to hypertension with complications, such as diabetes mellitus, kidney disease, and heart disease (Javadi et al., 2019; Susalit et al., 2011). The five trials involved 325 participants, 220 of which were female and 105 were male.

### Intervention

Trial participants were randomized regarding intervention and control groups. Olive leaf extract was given in 1,000 mg (Susalit et al., 2011; Wong et al., 2014) and 500 mg per day doses (Javadi et al., 2019; Perrinjaquet-Moccetti et al., 2008; Yaghoobzadeh et al., 2020). For the 1,000 mg per day dose, one trial used a combined formulation of 500 mg olive leaf extract with green coffee bean extract and beetroot tablet to be taken twice daily (Wong et al., 2014) and one trial used a 500 mg olive leaf extract tablet twice daily (Susalit et al., 2011). For the 500 mg per day dose, one trial used the olive leaf extract 500 mg tablet once daily (Perrinjaquet-Moccetti et al., 2008) and two trials used the 250 mg olive leaf extract tablet twice daily (Javadi et al., 2019; Yaghoobzadeh et al., 2020). Three trials used a placebo as a control (Javadi et al., 2019; Wong et al., 2014; Yaghoobzadeh et al., 2020). One trial advocated lifestyle modification to the control group (Perrinjaquet-Moccetti et al., 2008) and one trial gave an antihypertensive (captopril) to the control group (Susalit et al., 2011). In all five trials included, there was no information about harvesting area and seasons. Three trials mentioned olive leaf extract manufactured by Frutarom Switzerland Ltd (Perrinjaquet-Moccetti et al., 2008; Susalit et al., 2011; Wong et al., 2014). From the three trials, one trial mentioned that 500 mg of olive leaf extract comprised 16%–24% oleuropein and ≥30% other polyphenols (Wong et al., 2014). One trial stated that 500 mg olive leaf extract manufactured comprised 16–24% (m/m) oleuropein and the batch used had a content of 19.9% (m/m) (Susalit et al., 2011). One trial mentioned that characteristic components in the 500 mg olive leaf extract were 18–26% (m/m) oleuropein, 30–40% (m/m) polyphenols as well as verbascoside and luteolin-7-glucoside whereby, the batch used had oleuropein content of 20.8% (m/m) (Perrinjaquet-Moccetti et al., 2008). Two trials manufactured olive leaf extract by Barij medicinal plant research center, Kashan, Iran (Javadi et al., 2019; Yaghoobzadeh et al., 2020), whereby one trial mentioned that 250 mg olive leaf extract contains 16% oleuropein (Javadi et al., 2019) and another trial did not mention the components of olive leaf extract (Yaghoobzadeh et al., 2020).

### Outcomes

All five trials performed the intervention for a minimum of six weeks, and these were grouped according to three comparisons. The first comparison, which involved only one trial, compared a placebo with the 1,000 mg per day combined formulation of olive leaf extract, and it measured both primary outcomes and secondary outcomes (Wong et al., 2014). The secondary outcomes measured in the first comparison were the lipid profile and glucose metabolism. The second comparison, which involved three trials, compared the 500 mg per day olive leaf extract with a placebo or with no treatment (Javadi et al., 2019; Perrinjaquet-Moccetti et al., 2008; Yaghoobzadeh et al., 2020). In the second comparison, two trials measured both primary and secondary outcomes, whereby the secondary outcome was only the lipid profile (Perrinjaquet-Moccetti et al., 2008; Yaghoobzadeh et al., 2020). One trial in the second comparison measured only secondary outcomes, which were glucose metabolism, inflammatory markers, kidney and liver functions (Javadi et al., 2019). In the third comparison, which involved one trial, 1,000 mg per day of olive leaf extract was compared with an antihypertensive drug, and it measured both primary outcomes and secondary outcomes (Susalit et al., 2011). The secondary outcomes in the third comparison were the lipid profile, kidney and liver functions.

We were able to analyze two out of three trials from the second comparison as the two trials measured primary outcomes and lipid profile. Another trial in the second comparison only measures other secondary outcomes on which a review was performed. Meta-analysis was intended for the first and third comparison, but there were insufficient trials, thus only a review was performed.

### Risk of bias in included studies

**Figure 2:**
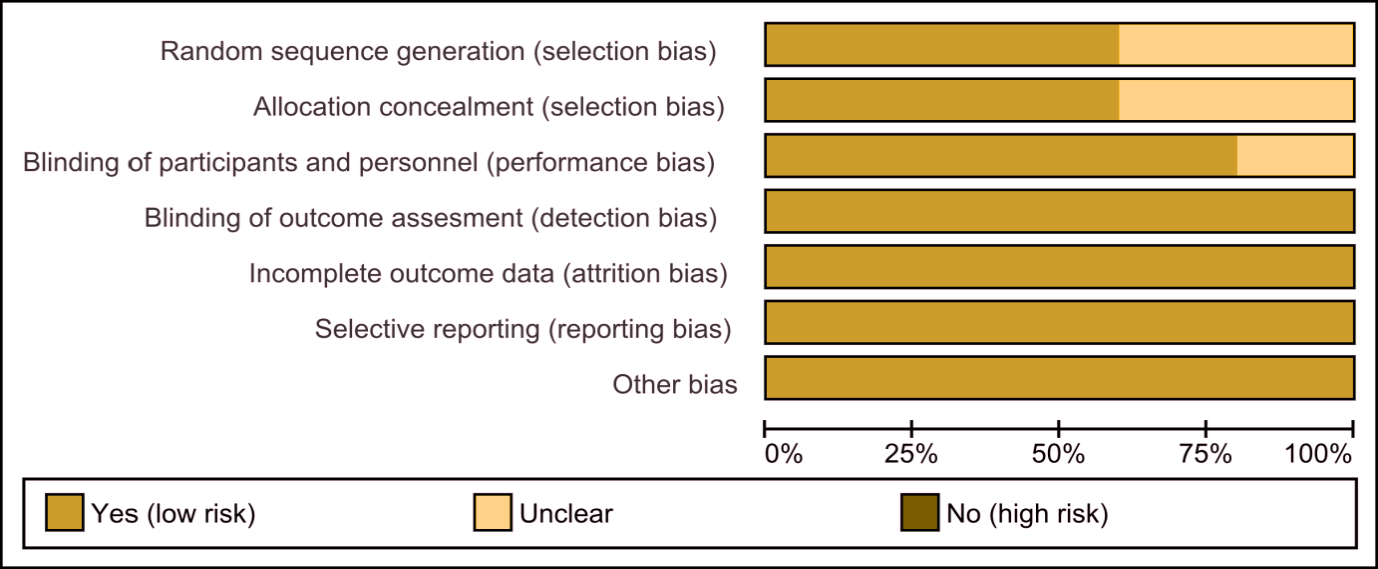
Risk of bias graph. Review authors’ judgements about each risk of bias item presented as percentages across all included studies.

**Figure 3:**
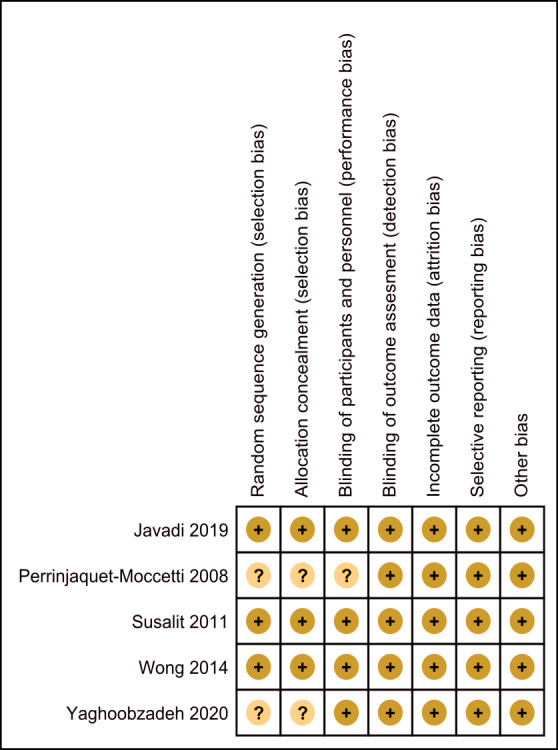
Risk of bias summary. Review authors’ judgements about each risk of bias item for each included study.

The assessment of the risk of bias is shown in Figs. 2 and 3. Figure 2 shows the proportion of studies that were assessed as having a low, high or unclear risk of bias with regard to each risk-of-bias indicator. Figure 3 shows the risk-of-bias indicators for individual studies.

#### Allocation

Three trials described the methods of randomization, which were computer-generated numbers (Javadi et al., 2019), randomization of numbers (Susalit et al., 2011), and the minimization method based on age, gender and BMI (Wong et al., 2014). The three trials were assessed as having a low risk of selection bias. One trial indicated randomization but did not describe the method (Yaghoobzadeh et al., 2020) and was assessed as having an unclear risk of selection bias. One trial stated that the participants, who were twins, were randomly assigned to different groups but did not describe the method (Perrinjaquet-Moccetti et al., 2008) and was assessed as having an unclear risk of selection bias.

#### Blinding

Three trials used matched drugs (Javadi et al., 2019; Wong et al., 2014; Yaghoobzadeh et al., 2020), thus based on the blinding of participants and personnel, it was assessed as having a low risk for performance bias. One trial stated that the drugs were given in a double-blind double-dummy fashion, in which dummies of each medication contained the same ingredients as the respective active preparations but without the active substance (Susalit et al., 2011), thus based on the blinding of participants and personnel, it was also assessed as having a low risk for performance bias. One trial divided 20 pairs of identical twins, with one of each pair in the control and intervention groups. Still, it did not describe the method of blinding (Perrinjaquet-Moccetti et al., 2008), so it was assessed as having an unclear risk for performance bias. The outcomes of all trials were objectively measured and were assessed as having a low risk of detection bias.

#### Incomplete outcome data

Two trials stated that they were missing data (Susalit et al., 2011; Wong et al., 2014), but they were almost balanced between groups. The missing data comprised 18 out of 90 participants in the olive leaf extract group, of which eight were due to non-compliance, six were due to ineffective therapy, one was due to an adverse effect and three were due to other reasons, whereas in the captopril group the missing data comprised 13 out of 89 participants, of which eight were due to non-compliance, two were due to ineffective therapy, one was a screened failure and two were due to other reasons (Susalit et al., 2011). In another trial, the missing data comprised one participant in the olive leaf extract combination group due to work commitments and one participant in the placebo group due to the commencement of antihypertensive medication (Wong et al., 2014). Three trials reported no missing data (Javadi et al., 2019; Perrinjaquet-Moccetti et al., 2008; Yaghoobzadeh et al., 2020). All five trials were assessed as having a low risk of attrition bias.

#### Selective reporting

All trials reported outcomes as specified in their methods sections (Javadi et al., 2019; Perrinjaquet-Moccetti et al., 2008; Susalit et al., 2011; Wong et al., 2014; Yaghoobzadeh et al., 2020).

### Effects of interventions

#### Comparison 1: Combined formulation with olive leaf extract vs placebo

Only one trial with a 1,000 mg per day dose of the combined formulation of olive leaf extract was available for comparison (Wong et al., 2014). For the primary outcomes, the combined formulation with olive leaf extract reported no significant changes in systolic and diastolic BP in comparison to the placebo.

For the secondary outcomes, the combined formulation with olive leaf extract reported no significant changes in TC, LDL, HDL, TG, FBS, insulin or HOMA-IR in comparison to the placebo. Overall, the quality of evidence shows a low certainty of evidence (Table 2).

**Table 2:** Summary of findings including GRADE quality assessment for comparison between combine formulation of olive leaf extract and placebo.

		**No. of participants**		Anticipated absolute effects^*^				
**Outcomes**	No. of participants (studies)	**placebo**	**combined formulation of 1,000 mg of olive leaf extract per day**	**MD**	**95% CI**	*P*-value	**Certainty of the evidence (GRADE)**	**Comments**
Systolic blood pressure (mmHg)	37 (1 RCT)	19	18	**0.54 lower**	−4.76 to 3.68	0.80	⊕⊕$○ $$○ $ LOW^a^^,^^b^	Risk of bias: not serious Inconsistency: serious^a^^,^^b^ indirectness: not serious; imprecision: serious^a^;
Diastolic blood pressure (mmHg)	37 (1 RCT)	19	18	**0.49 lower**	−2.75 to 1.77	0.67	⊕⊕$○ $$○ $ LOW^a^^,^^b^	Risk of bias: not serious Inconsistency: serious^a^^,^^b^; indirectness: not serious; imprecision: serious^a^;
Total cholesterol (mg/dl)	37 (1 RCT)	19	18	**9.66 higher**	−2.15 to 21.47	0.11	⊕⊕$○ $$○ $ LOW^a^^,^^b^	Risk of bias: not serious Inconsistency: serious^a^^,^^b^; indirectness: not serious; imprecision: serious^a^;
LDL (mg/dl)	37 (1 RCT)	19	18	**0**	−12.52 to 12.52	1.00	⊕⊕$○ $$○ $ LOW^a^^,^^b^	Risk of bias: not serious Inconsistency: serious^a^^,^^b^; indirectness: not serious; imprecision: serious^a^;
HDL (mg/dl)	37 (1 RCT)	19	18	**1.16 higher**	−1.55 to 3.87	0.40	⊕⊕$○ $$○ $ LOW^a^^,^^b^	Risk of bias: not serious Inconsistency: serious^a^^,^^b^; indirectness: not serious; imprecision: serious^a^;
TG (mg/dl)	37 (1 RCT)	19	18	**46.87 higher**	−0.38 to 94.12	0.05	⊕⊕$○ $$○ $ LOW^a^^,^^b^	Risk of bias: not serious Inconsistency: serious^a^^,^^b^; indirectness: not serious; imprecision: serious^a^;
HOMA-IR	37 (1 RCT)	19	18	**0.4 higher**	0.27 to 0.53	<0.001	⊕⊕$○ $$○ $ LOW^a^^,^^b^	Risk of bias: not serious Inconsistency: serious^a^^,^^b^; indirectness: not serious; imprecision: serious^a^;
Fasting blood glucose (mmol/l)	37 (1 RCT)	19	18	**0.01 higher**	−0.21 to 0.23	<0.93	⊕⊕$○ $$○ $ LOW^a^^,^^b^	Risk of bias: not serious Inconsistency: serious^a^^,^^b^; indirectness: not serious; imprecision: serious^a^;
Insulin (µu/ml)	37 (1 RCT)	19	18	**1.08 higher**	0.56 to 1.6	<0.001	⊕⊕$○ $$○ $ LOW^a^^,^^b^	Risk of bias: not serious Inconsistency: serious^a^^,^^b^; indirectness: not serious; imprecision: serious^a^;

**Notes.**

* **The risk in the intervention group** (and its 95% confidence interval) is based on the assumed risk in the comparison group and the **relative effect** of the intervention (and its 95% CI).

CI Confidence interval MD Mean difference

Explanations

a small population

b wide CI

**GRADE Working Group grades of evidence.**

**High certainty:** We are very confident that the true effect lies close to that of the estimate of the effect **Moderate certainty:** We are moderately confident in the effect estimate: The true effect is likely to be close to the estimate of the effect, but there is a possibility that it is substantially different.

**Low certainty:** Our confidence in the effect estimate is limited: The true effect may be substantially different from the estimate of the effect.

**Very low certainty:** We have very little confidence in the effect estimate: The true effect is likely to be substantially different from the estimate of effect.

#### Comparison 2: Olive leaf extract vs placebo or no treatment

**Figure 4:**
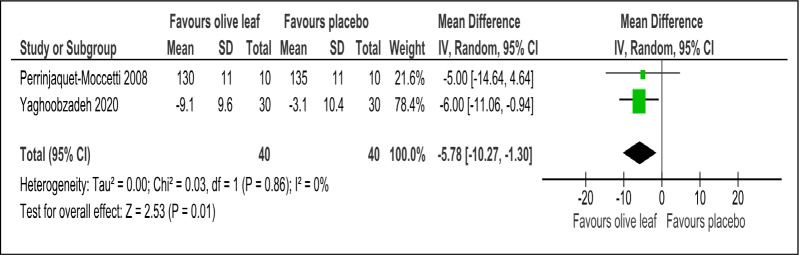
Forest plot of comparison 2: 500 mg per day olive leaf extract vs placebo or no treatment. Outcome: Systolic BP (mmHg).

**Figure 5:**
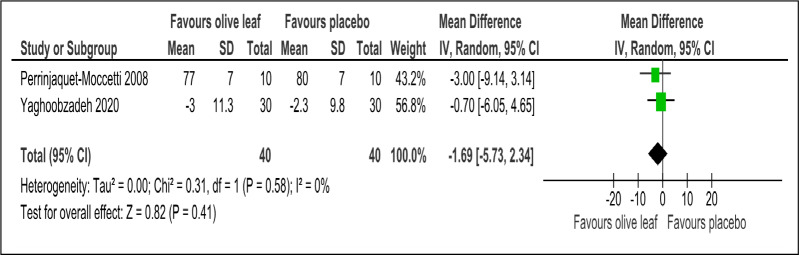
Forest plot of comparison 2: 500 mg per day olive leaf extract vs placebo or no treatment. Outcome: Diastolic BP (mmHg).

**Figure 6:**
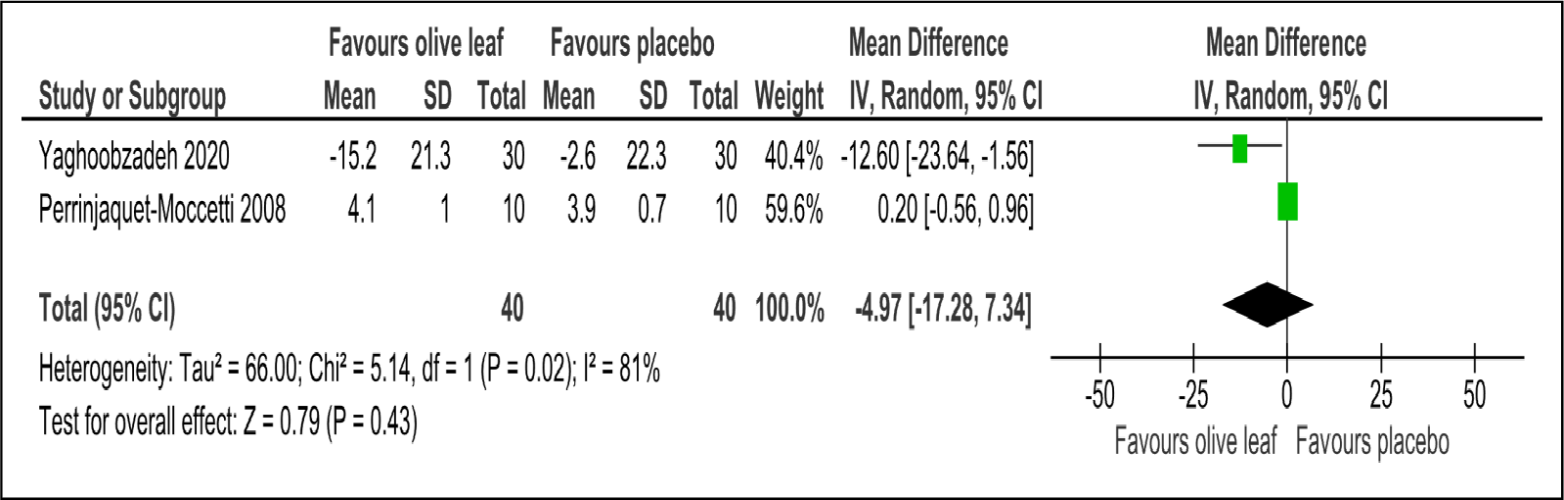
Forest plot of comparison 2: 500 mg per day olive leaf extract vs placebo or no treatment. Outcome: TC (mg/dl).

**Figure 7:**
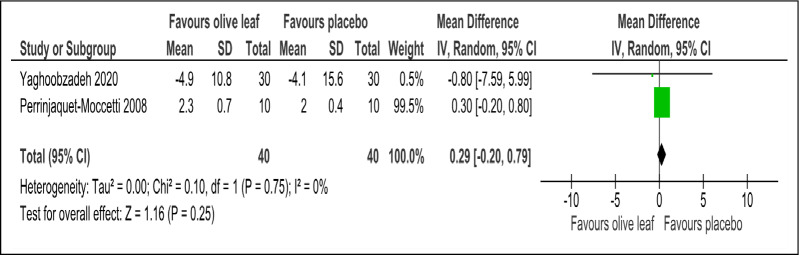
Forest plot of comparison 2: 500 mg per day olive leaf extract vs placebo or no treatment. Outcome: LDL (mg/dl).

**Figure 8:**
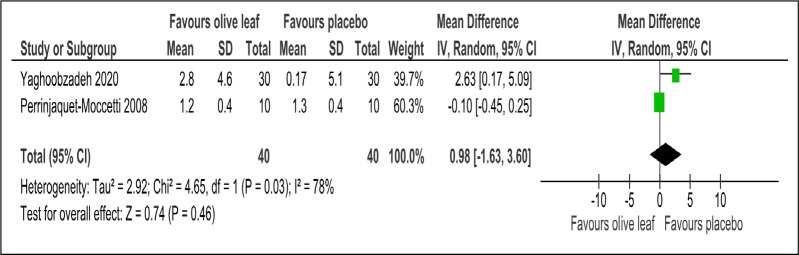
Forest plot of comparison 2: 500 mg per day olive leaf extract vs placebo or no treatment. Outcome: HDL (mg/dl).

There were two trials of a 500 mg per day olive leaf extract (Perrinjaquet-Moccetti et al., 2008; Yaghoobzadeh et al., 2020) that measured primary and secondary outcomes, while one trial of the 500 mg per day olive leaf extract (Javadi et al., 2019) measured only the secondary outcomes. For the primary outcomes, the olive leaf extract reported decreases in systolic BP (MD −5.78 mmHg, 95% CI [−10.27 to −1.30]; I^2^ = 0%, *p* = .01; two trials, 80 participants; low certainty of evidence) [Fig. 4 and Table 3] and no significant effect on diastolic BP in comparison to the control [Fig. 5 and Table 3]. For the secondary outcomes, the olive leaf extract reported no significant effect on TC [Fig. 6 and Table 3], LDL [Fig. 7 and Table 3], HDL [Fig. 8 and Table 3), FBS, HOMA-IR, ALT or AST in comparison to the control. However, the olive leaf extract reported decreases in IL-6 (MD −6.83 ng/L, 95% CI [−13.15 to −0.51], *p* = .03; one trial, 60 participants), IL-8 (MD −8.24 ng/L, 95% CI [−16.00 to −0.48], *p* = .04; one trial, 60 participants) and TNF-alpha (MD −7.40 ng/L, 95% CI [−13.23 to −1.57], *p* = .01; one trial, 60 participants) in comparison to the control. Overall, the quality of evidence showed low certainty (Table 3). We were unable to analyze any difference between the 500 mg, single dose per day olive leaf extract and 250 mg, twice daily dose olive leaf extract due to limited trials.

**Table 3:** Summary of findings including GRADE quality assessment for comparison between olive leaf extract and placebo or no treatment.

		No. of participants		Anticipated absolute effects ^*^				
**Outcomes**	No. of participants (studies)	**Placebo or no treatment**	**Olive leaf extract 500 mg per day dosage**	**MD**	**95% CI**	*P*-value	**Certainty of the evidence (GRADE)**	**Comments**
Systolic blood pressure (mmHg)	80 (2 RCTs)	40	40	**5.78 lower**	−10.27 to -1.3, I^2^= 0%	0.01	⊕⊕$○ $$○ $ LOW^a^	Risk of bias: not serious Inconsistency: serious^a^; indirectness: not serious; imprecision: serious^a^;
Diastolic blood pressure (mmHg)	80 (2 RCTs)	40	40	**1.69 lower**	−5.73 to 2.34, I^2^= 0%	0.41	⊕⊕$○ $$○ $ LOW^a^^,^^b^	Risk of bias: not serious Inconsistency: serious^a^^,^^b^; indirectness: not serious; imprecision: serious^a^;
Total cholesterol (mg/dl)	80 (2 RCTs)	40	40	**4.97 lower**	−17.28 to 7.34, I^2^= 81%	0.43	⊕⊕$○ $$○ $ LOW^a^^,^^b^	Risk of bias: not serious Inconsistency: serious^a^^,^^b^; indirectness: not serious; imprecision: serious^a^;
LDL (mg/dl)	80 (2 RCTs)	40	40	**0.29 higher**	−0.2 to 0.79, I^2^= 0%	0.25	⊕⊕$○ $$○ $ LOW^a^^,^^b^	Risk of bias: not serious Inconsistency: serious^a^^,^^b^; indirectness: not serious; imprecision: serious^a^;
HDL (mg/dl)	80 (2 RCTs)	40	40	**0.98 higher**	−1.63 to 3.6, I^2^= 78%	0.46	⊕⊕$○ $$○ $ LOW^a^^,^^b^	Risk of bias: not serious Inconsistency: serious^a^^,^^b^; indirectness: not serious; imprecision: serious^a^;
Fasting blood glucose (mmol/l)	60 (1 RCT)	30	30	**0.08 higher**	−0.25 to 0.4	0.65	⊕⊕$○ $$○ $ LOW^a^	Risk of bias: not serious Inconsistency: serious^a^; indirectness: not serious; imprecision: serious^a^;
HOMA-IR	60 (1 RCT)	30	30	**0.17 higher**	−0.17 to 0.51	0.33	⊕⊕$○ $$○ $ LOW^a^	Risk of bias: not serious Inconsistency: serious^a^; indirectness: not serious; imprecision: serious^a^;
Insulin (µu/ml)	60 (1 RCT)	30	30	**0.61 higher**	−0.84 to 2.06	0.41	⊕⊕$○ $$○ $ LOW^a^	Risk of bias: not serious Inconsistency: serious^a^; indirectness: not serious; imprecision: serious^a^;
creatinine (mg/dl)	60 (1 RCT)	30	30	**0.09 higher**	−0.18 to 0.36	0.52	⊕⊕$○ $$○ $ LOW^a^	Risk of bias: not serious Inconsistency: serious^a^; indirectness: not serious; imprecision: serious^a^;
ALT (U/L)	60 (1 RCT)	30	30	**0.3 higher**	−2.26 to 2.86	0.82	⊕⊕$○ $$○ $ LOW^a^	Risk of bias: not serious Inconsistency: serious^a^; indirectness: not serious; imprecision: serious^a^;
AST (U/L)	60 (1 RCT)	30	30	**0.57 higher**	−2 to 3.14	0.66	⊕⊕$○ $$○ $ LOW^a^	Risk of bias: not serious Inconsistency: serious^a^; indirectness: not serious; imprecision: serious^a^;
IL-6 (ng/L)	60 (1 RCT)	30	30	**6.83 lower**	−13.15 to −0.51	0.03	⊕⊕$○ $$○ $ LOW^a^	Risk of bias: not serious Inconsistency: serious^a^; indirectness: not serious; imprecision: serious^a^;
IL-8 (ng/L)	60 (1 RCT)	30	30	**8.24 lower**	−16 to -0.48	0.04	⊕⊕$○ $$○ $ LOW^a^	Risk of bias: not serious Inconsistency: serious^a^; indirectness: not serious; imprecision: serious^a^;
TNF-alpha (ng/L)	60 (1 RCT)	30	30	**7.4 lower**	−13.23 to -1.57	0.01	⊕⊕$○ $$○ $ LOW^a^	Risk of bias: not serious Inconsistency: serious^a^; indirectness: not serious; imprecision: serious^a^;

**Notes.**

* **The risk in the intervention group** (and its 95% confidence interval) is based on the assumed risk in the comparison group and the **relative effect** of the intervention (and its 95% CI). **CI:** Confidence interval; **MD:** Mean difference.

Explanations

a small population.

b wide CI.

**GRADE Working Group grades of evidence**

**High certainty:** We are very confident that the true effect lies close to that of the estimate of the effect

**Moderate certainty:** We are moderately confident in the effect estimate: The true effect is likely to be close to the estimate of the effect, but there is a possibility that it is substantially different

**Low certainty:** Our confidence in the effect estimate is limited: The true effect may be substantially different from the estimate of the effect

**Very low certainty:** We have very little confidence in the effect estimate: The true effect is likely to be substantially different from the estimate of effect

#### Comparison 3: Olive leaf extract vs antihypertensive drug

Only one trial with a 1,000 mg per day dose of olive leaf extract was available for comparison (Susalit et al., 2011). For the primary outcomes, the olive leaf extract reported no significant effect on systolic or diastolic BP. For the secondary outcomes, the olive leaf extract reported no significant effect on TC, HDL, TG, Creatinine or AST in comparison to captopril. However, the olive leaf extract reported a reduction in LDL (MD −6.00 mg/dl, 95% CI [−11.5 to −0.50]; *p* = .03; one trial, 148 participants) in comparison to captopril. Overall, the quality of evidence showed low certainty (Table 4).

## Discussion

This review was designed to include all RCTs to evaluate the effectiveness of olive leaf extract as a supplement on the cardiometabolic profile of patients with elevated blood pressure (prehypertension and hypertension). The five identified trials included in the review were divided into three comparisons for measuring primary and secondary outcomes. For the primary outcomes, the analysis was done on two trials that include 500 mg per day of olive leaf extract compared to the placebo or no treatment over eight weeks shows a reduction of systolic BP by 5.78 mmHg. This result is comparable with the findings of a systematic review and meta-analysis, which concluded that healthy dietary patterns, such as the Dietary Approaches to Stop Hypertension diet, Nordic diet, and Mediterranean diet, significantly lowered systolic BP by 4.26 mmHg (Ndanuko et al., 2016). However, the changes to diastolic BP were not significant. The other trials in our review did not support 1,000 mg olive leaf extract for reduction of BP compared to anti-hypertensives or placebo. The 1,000 mg dosage of olive leaf extract compared to captopril did not show significant changes to BP but show almost similar BP reduction to captopril and there were limited trials. The combined formulation of 1,000 mg of olive leaf extract compared to the placebo did not show significant changes to BP and there were also limited trials. In addition, subgroup analysis based on prehypertension vs hypertension and high dose (1,000 mg per day) vs low dose (500 mg per day) could not be performed due to the limited number of trials.

**Table 4:** Summary of findings including GRADE quality assessment for comparison between olive leaf extract and antihypertensive drugs.

		**No. of participants**		Anticipated absolute effects^*^				
**Outcomes**	No. of participants (studies)	**Captopril 12.5–25 mg**	**Olive leaf extract 1000 mg per day**	**MD**	**95% CI**	*P*-value	**Certainty of the evidence (GRADE)**	**Comments**
Systolic blood pressure (mmHg)	148 (1 RCT)	72	76	**2.2 higher**	−0.43 to 4.83	0.10	⊕⊕$○ $$○ $ LOW^a^^,^^b^	Risk of bias: not serious Inconsistency: serious^a^^,^^b^; indirectness: not serious; imprecision: serious^a^;
Diastolic blood pressure (mmHg)	148 (1 RCT)	72	76	**1.6 higher**	−0.13 to 3.33	0.07	⊕⊕$○ $$○ $ LOW^a^^,^^b^	Risk of bias: not serious Inconsistency: serious^a^^,^^b^; indirectness: not serious; imprecision: serious^a^;
Total cholesterol (mg/dl)	148 (1 RCT)	72	76	**6.3 lower**	−12.75 to 0.15	0.06	⊕⊕$○ $$○ $ LOW^a^^,^^b^	Risk of bias: not serious Inconsistency: serious^a^^,^^b^; indirectness: not serious; imprecision: serious^a^;
LDL (mg/dl)	148 (1 RCT)	72	76	**6 lower**	−11.5 to -0.5	0.03	⊕⊕$○ $$○ $ LOW^a^^,^^b^	Risk of bias: not serious Inconsistency: serious^a^^,^^b^; indirectness: not serious; imprecision: serious^a^;
HDL (mg/dl)	148 (1 RCT)	72	76	**1 higher**	−0.79 to 2.79	0.27	⊕⊕$○ $$○ $ LOW^a^^,^^b^	Risk of bias: not serious Inconsistency: serious^a^^,^^b^; indirectness: not serious; imprecision: serious^a^;
TG (mg/dl)	148 (1 RCT)	72	76	**10.6 lower**	−25.04 to 3.84	0.15	⊕⊕$○ $$○ $ LOW^a^^,^^b^	Risk of bias: not serious Inconsistency: serious^a^^,^^b^; indirectness: not serious; imprecision: serious^a^;
creatinine (mg/dl)	148 (1 RCT)	72	76	**0.02 higher**	−0.03 to 0.07	0.45	⊕⊕$○ $$○ $ LOW^a^^,^^b^	Risk of bias: not serious Inconsistency: serious^a^^,^^b^; indirectness: not serious; imprecision: serious^a^;
ALT (U/L)	148 (1 RCT)	72	76	**1.1 higher**	−1.95 to 4.15	0.48	⊕⊕$○ $$○ $ LOW^a^^,^^b^	Risk of bias: not serious Inconsistency: serious^a^^,^^b^; indirectness: not serious; imprecision: serious^a^;
AST (U/L)	148 (1 RCT)	72	76	**1.9 higher**	0.2 to 3.6	0.03	⊕⊕$○ $$○ $ LOW^a^^,^^b^	Risk of bias: not serious Inconsistency: serious^a^^,^^b^; indirectness: not serious; imprecision: serious^a^;

**Notes.**

* **The risk in the intervention group** (and its 95% confidence interval) is based on the assumed risk in the comparison group and the **relative effect** of the intervention (and its 95% CI).

CI Confidence interval MD Mean difference

Explanations

a small population

b wide CI

**GRADE Working Group grades of evidence**

**High certainty:** We are very confident that the true effect lies close to that of the estimate of the effect

**Moderate certainty:** We are moderately confident in the effect estimate: The true effect is likely to be close to the estimate of the effect, but there is a possibility that it is substantially different

**Low certainty:** Our confidence in the effect estimate is limited: The true effect may be substantially different from the estimate of the effect

**Very low certainty:** We have very little confidence in the effect estimate: The true effect is likely to be substantially different from the estimate of effect

Neither the 500 mg dosage of olive leaf extract (vs placebo or no treatment) nor the 1,000 mg combined formulation of olive leaf extract (vs placebo) showed any significant effects on the lipid profile. However, one trial found that the 1,000 mg olive leaf extract showed a significant reduction of LDL by 6 mg/dl compared to captopril though the lipid profile for the others reported no significant changes. For glucose metabolism, neither the 500 mg olive leaf extract nor the 1,000 mg combined formulation of olive leaf extract (compared to the placebo) showed any significant effects. For markers of inflammation, the 500 mg dosage of olive leaf extract showed a significant reduction of IL-6, IL-8 and TNF-alpha compared to the placebo. Concerning the safety of kidney and liver, there were no significant changes in creatinine, ALT or AST from either the 500 mg or the 1,000 mg olive leaf extract per day in comparison to the placebo or captopril, respectively.

### Applicability of evidence

In the global perspective, dietary risks were reported to be associated with 2.1 million cardiovascular deaths in the WHO European Region that accounts for 49.2% of CVD deaths and 22.4% of all deaths (Meier et al., 2019). Recognizing the increasing burden of non-communicable disease, WHO had emphasized the importance of dietary modifications as strategies to combat the non-communicable disease (Waxman, 2004). In Malaysia, the Clinical Practice Guideline on Primary and Secondary Prevention of CVD 2017s also emphasized that dietary habits have beneficial effects on cardiometabolic risk factors, such as weight, blood pressure, glucose metabolism, cholesterol, oxidative stress and inflammation. As there were ongoing research and interest in olive leaf extract, in the future it can act as a daily supplement to prevent CVD in addition to dietary modifications and lifestyle changes. Olive leaf, which originates from the olive tree, may have beneficial effects on cardiometabolic risk factors that had been used traditionally and contemporary globally as medicine which acts as folks remedy in the community for example to treat hypertension, diabetes and acts as an anti-inflammatory (Hashmi et al., 2015). However, in Malaysia there has been no reported use of olive leaf extract among the community.

In this review, we included both prehypertensive and hypertensive populations because of the increased risk of cardiovascular morbidity and mortality in comparison to the normotensive population (Zhang & Li, 2011). In addition, systolic BP is said to be superior to diastolic BP as a predictor of cardiovascular outcomes (Mourad, 2008). Furthermore, it was reported that, increased systolic BP was the leading risk factor for women and the second leading risk factor for men globally whereby, IHD was the largest source attributable to increased systolic BP, followed by hemorrhagic stroke and ischemic stroke (GBDRF Collaborators, 2017). The analysis of two trials has shown that 500 mg per day dose of olive leaf extract is beneficial for the reduction of systolic BP (around 5.78 mmHg). This is comparable to conventional perindopril monotherapy, as reported in the perindopril protection against recurrent stroke study, where perindopril monotherapy reduced systolic BP by five mmHg (PROGRESS, 2001). Furthermore, one review has shown a linear association between systolic BP and risk of cardiovascular disease and mortality, with the lowest risk at a systolic BP of 120 to 140 mmHg (Bundy et al., 2017). Only one trial found that LDL was reduced, with no other effects on the rest of the lipid profile, as the 1,000 mg dosage of olive leaf extract shows a reduction of LDL by 6 mg/dl in comparison to captopril. The reduction of LDL is important, as a reduction of LDL by 38.7 mg/dl ( one mmol/L) in a patient without known atherosclerotic cardiovascular disease will lead to predicted risk of major vascular events of 15% and of hard cardiovascular events (cardiovascular death, MI, or stroke) of 10% in the next 10 years, which indicates risk reductions of approximately 3.5% and 2.3%, respectively (Silverman et al., 2016). This review also shows that olive leaf extract confers some effects on the inflammatory markers involved in atherosclerosis. In terms of safety, this review shows that consuming olive leaf extract did not have a significant effect on creatinine, AST or ALT, which indicates a possible use for patients with liver and kidney disorders who have hypertension. Thus, there is a possible benefit from olive leaf extract in terms of modifying the cardiometabolic profile to reduce the risk of CVD.

### Quality of evidence

Applying the Cochrane methodology was a major strength of this systematic review, comprising a pre-published protocol, a non-restricted and up-to-date literature search, independent data extraction by at least two authors and risk of bias assessment leading to (Grading of Recommendations, Assessment, Development and Evaluation) GRADE evaluations of important outcomes. However, the findings of the review are limited by the small number of trials involved. Overall, there was low bias among the trial’s domains. There were two trials with an unclear risk of random sequence generation. There was no evidence of selective reporting bias. Attrition bias was low for all trials. Three of the trials declared funding from natural health manufacturers. We used random-effects meta-analysis, in which we encountered low to substantial heterogeneity in the analysis. For the substantial heterogeneity, we were unable to do sensitivity analysis due to the limited number of similar trials for comparison. Hence, the overall quality of evidence contributing to this review as assessed using the GRADE approach was low.

### Limitations

There were limited trials on olive leaf extracts that met the inclusion criteria for the review. They further differed in terms of the olive leaf extract and comparator formulation, which meant we could not combine trials for analysis. Furthermore, two trials were funded by olive leaf extract companies. The component analysis was mentioned in four out of five trials. None of the trials mentioned olive leaf harvesting area and season. Lipoprotein subfractions- sdLDL, VLDL and vascular inflammation marker CRP could not be evaluated. It was reported as a positive correlation between lipoprotein subfractions and inflammatory markers may contribute to increasing cardiovascular risk (Zhang et al., 2015). IL8 has been reported not to be associated with cardiovascular events in a recent cohort study (Moreno Velásquez et al., 2019). In the future, further similar trials are needed to ensure more concrete evidence.

## Conclusion

The pathophysiology of CVD is complex and multifactorial. Hypertension, hypercholesterolemia, inflammation, oxidative stress and atherosclerotic changes are some important risk parameters. The systematic review and meta-analysis of olive leaf extract benefit the cardiometabolic profile among prehypertensive and hypertensive groups. It was associated with a reduction in systolic BP for the 500 mg per day dosage. The 1,000 mg per day dose may be comparable to an anti-hypertensive medication in one trial. Individual trials in the review also show the reduction of LDL and inflammatory biomarkers. However, the trials were limited, and the overall quality of evidence was low. There were no effects shown on glucose metabolism, creatinine or liver transaminases (ALT and AST). Future researchers need to gather more evidence by producing similar and longer trials with clearer dosing of active ingredients (Oleuropein concentration), and more consistent measurement of BP, inflammatory markers and lipid profile.

##  Supplemental Information

10.7717/peerj.11173/supp-1 Supplemental Information 1 PRISMA checklist

10.7717/peerj.11173/supp-2 Supplemental Information 2 Data collected from Yaghoobzadeh et al. (2020)’s trial

10.7717/peerj.11173/supp-3 Supplemental Information 3 Data collected from Javadi et al. (2019)’s trial

10.7717/peerj.11173/supp-4 Supplemental Information 4 Data collected from Wong et al. (2014)’s trial

10.7717/peerj.11173/supp-5 Supplemental Information 5 Data collected from Susalit et al. (2011)’s trial

10.7717/peerj.11173/supp-6 Supplemental Information 6 Data collected from Tania Perrinjaquet-Moccetti et al. (2008)’s trial

10.7717/peerj.11173/supp-7 Supplemental Information 7 Rationale

## Additional Information and Declarations

### Data Availability

The following information was supplied regarding data availability: Raw data are available in the Supplementary Files.
