# Supplementary material for: Olive leaf extract effect on cardiometabolic profile among adults with prehypertension and hypertension: a systematic review and meta-analysis
Source: PeerJ. 2021 Apr 7;9:e11173. doi: 10.7717/peerj.11173 (PMC8035902; doi:10.7717/peerj.11173)
Supplement: Supplemental Information 2 [file peerj-09-11173-s002.docx]

***Study Eligibility & Data Collection Form***

***General Information***

| **Study ID**  *(e.g. author name, year)* | Hayedeh Yaghoobzadeh, 2020 |
| --- | --- |
| **Form completed by** | Muhammad Asyraf Bin Ismail |
| **Study author contact details** | asyraf88fm@gmail.com |
| **Publication type**  *(e.g. full report, abstract, letter)* | Full Report |
| **List of included publications** |  |
| **References of similar trial*** |  |

*This is when the authors published the same study in several reports. All these references to a similar trial should be linked under one *Study ID* in RevMan.

***Study eligibility***

|  | Yes | No | Unclear | Further details |
| --- | --- | --- | --- | --- |
| **RCT/Quasi/CCT** | ***/*** |  |  | randomised, double-blind, placebo-controlled cross-over trial |
| **Relevant participants** | ***/*** |  |  |  |
| **Relevant interventions** | ***/*** |  |  |  |
| **Relevant outcomes*** | ***/*** |  |  |  |

*Include only if the presence of outcomes form the inclusion criterion

If the above answers are ‘YES’, proceed to Section 1.

If any of the above answers are ‘NO*’, record below the information for ‘Excluded studies’

| Reason(s) for exclusion |
| --- |
|  |

Section 1. Characteristics of included studies

This section is to be completed by only one reviewer. State initials: MAI

| **METHODS** | **Descriptions as stated in paper** |
| --- | --- |
| **Aim of study** *(e.g. efficacy, equivalence, pragmatic)* | To evaluate the effects of olive leaf extract (OLE), Olea europaea L., on cardiometabolic parameters and biomarkers of oxidative stress in patients with essential hypertension |
| **Design** *(e.g. parallel, crossover, cluster)* | randomized double-blind placebo controlled clinical trial |
| **Unit of allocation**  *(by individuals, cluster/ groups or body parts)* | Individuals |
| **Start & end dates** | 2017, no specific date was provided |
| **Total study duration** | 12 weeks |
| **Sources of funding**  *(including role of funders)* | Deputy for Research and Technology of the Qazvin University of Medical Sciences |
| **Possible conflicts of interest**  *(for study authors)* | no competing interest exists |

| **PARTICIPANTS** | **Description**  *(include information for each intervention or comparison group)* |
| --- | --- |
| **Population description**  *(Company/companies; occupation)* | Hypertensive patient |
| **Setting**  *(including location (city, state, country) and single centre / multicenter)* | Qazvin, Iran  Single center- cardiovascular clinic of Bu-Ali Sina Hospital affiliated to Qazvin University of Medical Sciences |
| **Inclusion criteria** | patients with essential hypertension aged 30-60 |
| **Exclusion criteria** | Not stated |
| **Method of recruitment of participants** *(e.g. phone, mail, clinic patients, voluntary)* | 60 patients referred to the cardiovascular clinic of Bu-Ali Sina Hospital affiliated to Qazvin University of Medical Sciences in Iran |
| **Total no. randomised** | 60 |
| **Clusters**  *(if applicable, no., type, no. people per cluster)* | None |
| **No. randomised per group**  *(specify whether no. people or clusters)* | Intervention: 30  Control: 30 |
| **No. missing**  *(if overall, e.g. exclusions & withdrawals, whether or not missing from analysis)* | Intervention: 0  Control: 0 |
| **Reasons missing** | Intervention: None  Control: None |
| **Baseline imbalances** |  |
| **Age** | Olive leaf extract: 52.9±10.3  Placebo: 57.9±10.8 |
| **Sex (proportion)** | Male: 30 Female 30 |
| **Race/Ethnicity** | Not stated |
| **Other relevant sociodemographics** | None |
| **Subgroups measured** *(eg split by age or sex)* | None |
| **Subgroups reported** | None |

Section 2. Risk of bias assessment

We recommend you refer to and use the method described in the Cochrane Handbook.

This section is completed by two reviewers. State initials: (i) MAI (ii) NMN

| **Domain** | **Risk of bias** | **Support for judgement**  *(include direct quotes where available with explanatory comments)* | **Location in text or source** *(page, table)* |
| --- | --- | --- | --- |
|  | Low/High/Unclear |  |  |
| **Random sequence generation**  *(selection bias)* | Unclear | Allocation based on randomisation | 373 |
| **Allocation concealment**  *(selection bias)* | Unclear | Participants were assigned to matched groups of OLE and placebo. Method not explained | 373 |
| **Blinding of participants and personnel**  *(performance bias)* | Low | randomized double blind | 373 |
| **Blinding of outcome assessment**  *(detection bias)* | Low | Objective outcome unlikely to be influenced. |  |
| **Incomplete outcome data**  *(attrition bias)* | Low | It doesn’t state any missing participants | 373 |
| **Selective outcome reporting**  *(reporting bias)* | Low | All outcomes were reported | Table 2 |
| **Other bias** | Low |  |  |

Random sequence generation = Process used to assign people into intervention and control groups

Allocation concealment = Process used to prevent foreknowledge of group assignment in a RCT

Blinding of participants and personnel = Presence or absence of blinding for participants and health personnel

Blinding of outcome assessment = presence or absence of blinding for assessment of outcome

Incomplete outcome data = application of intention-to-treat analysis is one in which all the participants in a trial are analysed according to the intervention to which they were allocated

Selective outcome reporting = Selection of a subset of the original variables recorded

***Section 3. Intervention groups***

This section is completed by two reviewers. State initials: (i) MAI (ii) NMN

| **Outcomes relevant to your review**  *(Copy and paste from ‘Types of outcome measures’)* | **Reported in paper**  *(Yes / No)* | **Outcome definition** *(with diagnostic criteria if relevant)* | **Unit of measurement & tool**  *(if relevant)* | **Reanalysis required?** *(specify)* |
| --- | --- | --- | --- | --- |
| Systolic blood pressure | Yes | Changes in clinical SBP | mmHg |  |
| Diastolic blood pressure | Yes | Changes in clinical DBP | mmHg |  |
| Lipid profile | Yes | 1) Total cholesterol  2) LDL  3) HDL  4) TG | mg/dl  mg/dl  mg/dl  mg/dl |  |
| Inflammatory markers for CVD | No | 1) IL-6  2) IL-8  3) TNF-alpha | ng/L  ng/L  ng/L |  |
| Glucose metabolism | No | 1) Fasting glucose  2) Insulin  3) HOMA-IR (insulin resistance) | mmol/L  µu/ml  no unit |  |
| Safety | No | 1) Creatinine  2) AST  3) ALT | mg/dl  U/L  U/L |  |
| Outcome 7 |  |  |  |  |
| Outcome 8 |  |  |  |  |

***Section 4. Data and analysis***

| **DICHOTOMOUS OUTCOME** | Intervention group | | Control group | |
| --- | --- | --- | --- | --- |
|  | Number of events | Number of participants | Number of events | Number of participants |
|  |  |  |  |  |
|  |  |  |  |  |
|  |  |  |  |  |
|  |  |  |  |  |
|  |  |  |  |  |
|  |  |  |  |  |

State details if outcomes were only described in text or figures.

| **CONTINUOUS OUTCOME** | Unit of measurement | Intervention group | | Control group | |
| --- | --- | --- | --- | --- | --- |
|  |  | n | Mean (SD) | n | Mean (SEM) |
| Systolic blood pressure | mmHg | 30 | - 9.1± 9.6 | **30** | - 3.1 ± 10.4 |
| Diastolic blood pressure | mmHg | 30 | - 3 ±11/3 | **30** | - 2.3 ± 9.8 |
| Lipid profile (TC) | mg/dl | 30 | - 15.2± 21.3 | **30** | - 2.6 ± 22.3 |
| Lipid profile (LDL) | mg/dl | 30 | - 4.9 ± 10.8 | **30** | -4.1 ± 15.6 |
| Lipid profile (HDL) | mg/dl | 30 | 2.8 ± 4.6 | **30** | 0.17 ± 5.1 |
| Lipid profile (TG) | mg/dl | 30 | -6.5 ± 29.2 | **30** | - 2 ± 32.9 |

State details if outcomes were only described in text or figures.

***Section 5. Other information***

|  | **Description as stated in paper** |
| --- | --- |
| **Key conclusions of study authors** | OLE intake for 12 weeks had beneficial effects on some types of cardiometabolic and oxidative stress biomarkers in hypertensive patients |
| **Results that you calculated using a formula** | None |
| **References to other relevant studies**  *(Did this report include any references to unpublished data from potentially eligible trials not already identified for this review? If yes, give list contact name and details)* | No |
| **Correspondence required for further study information** *(from whom, what and when)* | - |

**Sources:**

Higgins JPT, Green S (editors). Cochrane Handbook for Systematic Reviews of Interventions Version 5.1.0 [updated March 2011]. The Cochrane Collaboration, 2011.Available from www.cochrane-handbook.org.
