# Supplementary material for: Olive leaf extract effect on cardiometabolic profile among adults with prehypertension and hypertension: a systematic review and meta-analysis
Source: PeerJ. 2021 Apr 7;9:e11173. doi: 10.7717/peerj.11173 (PMC8035902; doi:10.7717/peerj.11173)
Supplement: Supplemental Information 3 [file peerj-09-11173-s003.doc]

***Study Eligibility & Data Collection Form***

***General Information***

| **Study ID**  *(e.g. author name, year)* | Hamidreza Javadi, 2019 |
| --- | --- |
| **Form completed by** | Muhammad Asyraf Bin Ismail |
| **Study author contact details** | asyraf88fm@gmail.com |
| **Publication type**  *(e.g. full report, abstract, letter)* | Full Report |
| **List of included publications** |  |
| **References of similar trial*** |  |

*This is when the authors published the same study in several reports. All these references to a similar trial should be linked under one *Study ID* in RevMan.

***Study eligibility***

|  | Yes | No | Unclear | Further details |
| --- | --- | --- | --- | --- |
| **RCT/Quasi/CCT** | ***/*** |  |  |  |
| **Relevant participants** | ***/*** |  |  |  |
| **Relevant interventions** | ***/*** |  |  |  |
| **Relevant outcomes*** | ***/*** |  |  |  |

*Include only if the presence of outcomes form the inclusion criterion

If the above answers are ‘YES’, proceed to Section 1.

If any of the above answers are ‘NO*’, record below the information for ‘Excluded studies’

| Reason(s) for exclusion |
| --- |
|  |

Section 1. Characteristics of included studies

This section is to be completed by only one reviewer. State initials: ……

| **METHODS** | **Descriptions as stated in paper** |
| --- | --- |
| **Aim of study** *(e.g. efficacy, equivalence, pragmatic)* | To determine effects of olive leaf extract on metabolic response, liver and kidney functions and inflammatory biomarkers in hypertensive patients |
| **Design** *(e.g. parallel, crossover, cluster)* | randomized double-blind placebo controlled clinical trial |
| **Unit of allocation**  *(by individuals, cluster/ groups or body parts)* | Groups |
| **Start & end dates** | December 2017-November 2018 |
| **Total study duration** | 12 weeks |
| **Sources of funding**  *(including role of funders)* | Research and technology affairs affiliated to Qazvin University of Medical Sciences, Qazvin, Iran |
| **Possible conflicts of interest**  *(for study authors)* | no competing interest exists |

| **PARTICIPANTS** | **Description**  *(include information for each intervention or comparison group)* |
| --- | --- |
| **Population description**  *(Company/companies; occupation)* | Hypertensive patients |
| **Setting**  *(including location (city, state, country) and single centre / multicenter)* | Qazvin, central region of Iran  Multicenter- Qazvin University of Medical Sciences (QUMS) and Booali-Sina hospital laboratory affiliated to QUMS |
| **Inclusion criteria** | Patients with hypertension |
| **Exclusion criteria** | Hypertensive patients who had complications such as: diabetes, kidney disease and hypo and/or hyperthyroidism were excluded |
| **Method of recruitment of participants** *(e.g. phone, mail, clinic patients, voluntary)* | health and clinical centers affiliated to Qazvin University of Medical Sciences |
| **Total no. randomised** | 60 |
| **Clusters**  *(if applicable, no., type, no. people per cluster)* | None |
| **No. randomised per group**  *(specify whether no. people or clusters)* | Intervention: 30  Control: 30 |
| **No. missing**  *(if overall, e.g. exclusions & withdrawals, whether or not missing from analysis)* | Intervention: 0  Control: 0 |
| **Reasons missing** | Intervention:  Control: |
| **Baseline imbalances** |  |
| **Age** | OLE: 53.8±8.0  Placebo: 55.6±8.8 |
| **Sex (proportion)** | OLE: Male- 14 Female-16  Placebo: Male- 13 Female 17 |
| **Race/Ethnicity** | Not stated |
| **Other relevant sociodemographics** | None |
| **Subgroups measured** *(eg split by age or sex)* | None |
| **Subgroups reported** | None |

Section 2. Risk of bias assessment

We recommend you refer to and use the method described in the Cochrane Handbook.

This section is completed by two reviewers. State initials: (i)…… (ii) ……

| **Domain** | **Risk of bias** | **Support for judgement**  *(include direct quotes where available with explanatory comments)* | **Location in text or source** *(page, table)* |
| --- | --- | --- | --- |
|  | Low/High/Unclear |  |  |
| **Random sequence generation**  *(selection bias)* | Low | Using computer generated random numbers | 343 |
| **Allocation concealment**  *(selection bias)* | Low | Randomly allocated | 343 |
| **Blinding of participants and personnel**  *(performance bias)* | low | Participants: The placebo and olive leaf extract were design as tablets | 343 |
| **Blinding of outcome assessment**  *(detection bias)* | Low | Objective outcome unlikely to be influenced |  |
| **Incomplete outcome data**  *(attrition bias)* | Low | No missing data and lost to follow up |  |
| **Selective outcome reporting**  *(reporting bias)* | Low | All outcomes were reported |  |
| **Other bias** | Low |  |  |

Random sequence generation = Process used to assign people into intervention and control groups

Allocation concealment = Process used to prevent foreknowledge of group assignment in a RCT

Blinding of participants and personnel = Presence or absence of blinding for participants and health personnel

Blinding of outcome assessment = presence or absence of blinding for assessment of outcome

Incomplete outcome data = application of intention-to-treat analysis is one in which all the participants in a trial are analysed according to the intervention to which they were allocated

Selective outcome reporting = Selection of a subset of the original variables recorded

***Section 3. Intervention groups***

This section is completed by two reviewers. State initials: (i)…… (ii) NMN

| **Outcomes relevant to your review**  *(Copy and paste from ‘Types of outcome measures’)* | **Reported in paper**  *(Yes / No)* | **Outcome definition** *(with diagnostic criteria if relevant)* | **Unit of measurement & tool**  *(if relevant)* | **Reanalysis required?** *(specify)* |
| --- | --- | --- | --- | --- |
| Systolic blood pressure | No | Changes in clinical SBP | mmHg |  |
| Diastolic blood pressure | No | Changes in clinical DBP | mmHg |  |
| Lipid profile | No | 1) Total cholesterol  2) LDL  3) HDL  4) TG | mg/dl  mg/dl  mg/dl  mg/dl |  |
| Inflammatory markers for CVD | Yes | 1) IL-6  2) IL-8  3) TNF-alpha | ng/L  ng/L  ng/L |  |
| Glucose metabolism | Yes | 1) Fasting glucose  2) Insulin  3) HOMA-IR (insulin  resistance) | mmol/L  µu/ml  no unit |  |
| Safety | Yes | 1) Creatinine  2) AST  3) ALT | mg/dl  U/L  U/L |  |
| Outcome 7 |  |  |  |  |
| Outcome 8 |  |  |  |  |

***Section 4. Data and analysis***

| **DICHOTOMOUS OUTCOME** | Intervention group | | Control group | |
| --- | --- | --- | --- | --- |
|  | Number of events | Number of participants | Number of events | Number of participants |
|  |  |  |  |  |
|  |  |  |  |  |
|  |  |  |  |  |
|  |  |  |  |  |
|  |  |  |  |  |
|  |  |  |  |  |

State details if outcomes were only described in text or figures.

| **CONTINUOUS OUTCOME** | Unit of measurement | Intervention group | | Control group | |
| --- | --- | --- | --- | --- | --- |
|  |  | n | Mean (SD) | n | Mean (SD) |
| Inflammatory markers for CVD- IL6 | ng/L | 30 | **-**5.9±13.4 | **30** | 0.93±11.5 |
| Inflammatory markers for CVD- IL8 | ng/L | 30 | -8.2±17.5 | **30** | 0.04±12.8 |
| Inflammatory markers for CVD- TNF- α | ng/L | 30 | -8.5±10.9 | **30** | -1.10±12.1 |
| Glucose metabolism (Fasting glucose) | mmo/l | 30 | 0.105± 0.68 | **30** | 0.029±0.6 |
| Glucose metabolism (Insulin) | µu/ml | 30 | 0.48±3.1 | **30** | -0.13±2.6 |
| Glucose metabolism (HOMA-IR)- homeostatic model of assessment for insulin resistance | No Unit | 30 | **0.15±0.66** | **30** | **-0.02±0.7** |
| Safety (creatinine) | mg/dl | 30 | 0.12±0.75 | **30** | 0.03±0.14 |
| Safety (ALT)-liver function | U/L | 30 | 0.73±5.3 | **30** | 0.43±4.8 |
| Safety (AST)-liver function | U/L | 30 | 0.37±5.9 | **30** | **-0.20±4.1** |

State details if outcomes were only described in text or figures.

***Section 5. Other information***

|  | **Description as stated in paper** |
| --- | --- |
| **Key conclusions of study authors** | This study discovered that this formulated tablet of OLE with this dose had no inverse effect on glucose metabolism related parameters and also on liver and kidney functions, but significantly improved inflammatory biomarkers (IL-6, IL-8 and TNF-α) |
| **Results that you calculated using a formula** | Convert fasting blood glucose unit from mg/dl to mmol/l |
| **References to other relevant studies**  *(Did this report include any references to unpublished data from potentially eligible trials not already identified for this review? If yes, give list contact name and details)* |  |
| **Correspondence required for further study information** *(from whom, what and when)* |  |

**Sources:**

Higgins JPT, Green S (editors). Cochrane Handbook for Systematic Reviews of Interventions Version 5.1.0 [updated March 2011]. The Cochrane Collaboration, 2011.Available from www.cochrane-handbook.org.
