# Supplementary material for: Olive leaf extract effect on cardiometabolic profile among adults with prehypertension and hypertension: a systematic review and meta-analysis
Source: PeerJ. 2021 Apr 7;9:e11173. doi: 10.7717/peerj.11173 (PMC8035902; doi:10.7717/peerj.11173)
Supplement: Supplemental Information 4 [file peerj-09-11173-s004.doc]

***Study Eligibility & Data Collection Form***

***General Information***

| **Study ID**  *(e.g. author name, year)* | Rachel H.X. Wong, 2014 |
| --- | --- |
| **Form completed by** | Muhammad Asyraf Bin Ismail |
| **Study author contact details** | asyraf88fm@gmail.com |
| **Publication type**  *(e.g. full report, abstract, letter)* | Full Report |
| **List of included publications** |  |
| **References of similar trial*** |  |

*This is when the authors published the same study in several reports. All these references to a similar trial should be linked under one *Study ID* in RevMan.

***Study eligibility***

|  | Yes | No | Unclear | Further details |
| --- | --- | --- | --- | --- |
| **RCT/Quasi/CCT** | ***/*** |  |  | randomised, double-blind, placebo-controlled cross-over trial |
| **Relevant participants** | ***/*** |  |  |  |
| **Relevant interventions** | ***/*** |  |  |  |
| **Relevant outcomes*** | ***/*** |  |  |  |

*Include only if the presence of outcomes form the inclusion criterion

If the above answers are ‘YES’, proceed to Section 1.

If any of the above answers are ‘NO*’, record below the information for ‘Excluded studies’

| Reason(s) for exclusion |
| --- |
|  |

Section 1. Characteristics of included studies

This section is to be completed by only one reviewer. State initials: ……

| **METHODS** | **Descriptions as stated in paper** |
| --- | --- |
| **Aim of study** *(e.g. efficacy, equivalence, pragmatic)* | Extracts of olive leaf, green coffee bean and beetroot may deliver cardiovascular benefits. This study sought to evaluate the effects of regularly consuming a combination of these extracts on blood pressure (BP), arterial compliance, blood lipids, blood glucose and insulin sensitivity |
| **Design** *(e.g. parallel, crossover, cluster)* | randomized double-blind placebo controlled clinical trial |
| **Unit of allocation**  *(by individuals, cluster/ groups or body parts)* | Individuals |
| **Start & end dates** | Between September and December 2013 |
| **Total study duration** | 12 weeks, but only result till week 6 before crossover was taken to be included in the study |
| **Sources of funding**  *(including role of funders)* | HealthWorld Ltd |
| **Possible conflicts of interest**  *(for study authors)* | no competing interest exists |

| **PARTICIPANTS** | **Description**  *(include information for each intervention or comparison group)* |
| --- | --- |
| **Population description**  *(Company/companies; occupation)* | Prehypertension and hypertensive patient |
| **Setting**  *(including location (city, state, country) and single centre / multicenter)* | Hunter region of New South Wales, Australia  Single center- Hunter Medical Research Institute, Clinical Nutrition Research Centre, University of Newcastle |
| **Inclusion criteria** | 18–80 years, body mass index (BMI) between 20 and 35 kg/m2  BP recorded at baseline/screening Visit 1 between 130–160 mmHg systolic and 85–100 mmHg diastolic |
| **Exclusion criteria** | smokers or taking nicotine therapy, taking antihypertensive medication or insulin, pregnant or currently breastfeeding, unwilling to wear the ABP monitor or undergo BP monitoring for 24 h, unwilling to maintain habitual diet and physical activity during the intervention, currently consuming dietary supplements containing extracts of olive leaf, green coffee bean or beet |
| **Method of recruitment of participants** *(e.g. phone, mail, clinic patients, voluntary)* | Volunteers were recruited from the general public via the Hunter Medical Research Institute research volunteer registry and public media announcements |
| **Total no. randomised** | 37 |
| **Clusters**  *(if applicable, no., type, no. people per cluster)* | None |
| **No. randomised per group**  *(specify whether no. people or clusters)* | Intervention: 19  Control: 18 |
| **No. missing**  *(if overall, e.g. exclusions & withdrawals, whether or not missing from analysis)* | Intervention: 1  Control: 1 |
| **Reasons missing** | Intervention: withdrew due to work commitments  Control: placed on medication |
| **Baseline imbalances** |  |
| **Age** | 58.5 ± 10.7 |
| **Sex (proportion)** | Male: 20 Female 17 |
| **Race/Ethnicity** | Not stated |
| **Other relevant sociodemographics** | None |
| **Subgroups measured** *(eg split by age or sex)* | None |
| **Subgroups reported** | None |

Section 2. Risk of bias assessment

We recommend you refer to and use the method described in the Cochrane Handbook.

This section is completed by two reviewers. State initials: (i)…… (ii) ……

| **Domain** | **Risk of bias** | **Support for judgement**  *(include direct quotes where available with explanatory comments)* | **Location in text or source** *(page, table)* |
| --- | --- | --- | --- |
|  | Low/High/Unclear |  |  |
| **Random sequence generation**  *(selection bias)* | Low | Allocation based on randomisation by minimisation method (age, gender and BMI) | 4883 |
| **Allocation concealment**  *(selection bias)* | Low | Independent investigator randomised participants to treatments and assigned container code numbers | 4884 |
| **Blinding of participants and personnel**  *(performance bias)* | Low | The olive leaf formulation and placebo tablets were identical in appearance, was dispensed in sealed white opaque containers, identifiable by code numbers. An independent investigator randomised participants to treatments and assigned container code numbers. The product was safely stored in a locked and limited access area. Trial investigators remained blinded until all data analysis been performed. | 4884 |
| **Blinding of outcome assessment**  *(detection bias)* | Low | Comment: Objective outcome unlikely to be influenced. Trial investigators remained blinded until all data analysis had been performed | 4884 |
| **Incomplete outcome data**  *(attrition bias)* | Low | Participants missing between both groups balanced, 1 from active group and 1 from placebo | 4886 |
| **Selective outcome reporting**  *(reporting bias)* | Low | All outcome for 6 weeks before cross-over was reported and attached on supplementary file |  |
| **Other bias** | Low |  |  |

Random sequence generation = Process used to assign people into intervention and control groups

Allocation concealment = Process used to prevent foreknowledge of group assignment in a RCT

Blinding of participants and personnel = Presence or absence of blinding for participants and health personnel

Blinding of outcome assessment = presence or absence of blinding for assessment of outcome

Incomplete outcome data = application of intention-to-treat analysis is one in which all the participants in a trial are analysed according to the intervention to which they were allocated

Selective outcome reporting = Selection of a subset of the original variables recorded

***Section 3. Intervention groups***

This section is completed by two reviewers. State initials: (i)…… (ii) NMN

| **Outcomes relevant to your review**  *(Copy and paste from ‘Types of outcome measures’)* | **Reported in paper**  *(Yes / No)* | **Outcome definition** *(with diagnostic criteria if relevant)* | **Unit of measurement & tool**  *(if relevant)* | **Reanalysis required?** *(specify)* |
| --- | --- | --- | --- | --- |
| Systolic blood pressure | Yes | Changes in clinical SBP | mmHg |  |
| Diastolic blood pressure | Yes | Changes in clinical DBP | mmHg |  |
| Lipid profile | Yes | 1) Total cholesterol  2) LDL  3) HDL  4) TG | mg/dl  mg/dl  mg/dl  mg/dl |  |
| Inflammatory markers for CVD | No | 1) IL-6  2) IL-8  3) TNF-alpha | ng/L  ng/L  ng/L |  |
| Glucose metabolism | Yes | 1) Fasting glucose  2) Insulin  3) HOMA-IR (insulin  resistance) | mmol/L  µu/ml  no unit |  |
| Safety | No | 1) Creatinine  2) AST  3) ALT | mg/dl  U/L  U/L |  |
| Outcome 7 |  |  |  |  |
| Outcome 8 |  |  |  |  |

***Section 4. Data and analysis***

| **DICHOTOMOUS OUTCOME** | Intervention group | | Control group | |
| --- | --- | --- | --- | --- |
|  | Number of events | Number of participants | Number of events | Number of participants |
|  |  |  |  |  |
|  |  |  |  |  |
|  |  |  |  |  |
|  |  |  |  |  |
|  |  |  |  |  |
|  |  |  |  |  |

State details if outcomes were only described in text or figures.

| **CONTINUOUS OUTCOME** | Unit of measurement | Intervention group | | Control group | |
| --- | --- | --- | --- | --- | --- |
|  |  | n | Mean (SD) | n | Mean (SD) |
| Systolic blood pressure | mmHg | 19 | 1.68 ± 7.06 | **18** | 2.22 ± 6.02 |
| Diastolic blood pressure | mmHg | 19 | 0.68 ± 3.48 | **18** | 1.17 ± 3.52 |
| Lipid profile (TC) | mg/dl | 19 | 7.34 ± 11.7 | **18** | −2.32 ± 22.9 |
| Lipid profile (LDL) | mg/dl | 19 | 4.25 ± 11.7 | **18** | 4.25 ± 24.6 |
| Lipid profile (HDL) | mg/dl | 19 | 1.93 ± 3.3 | **18** | 0.77 ± 4.9 |
| Lipid profile (TG) | mg/dl | 19 | 32.77 ± 92.4 | **18** | −14.1 ± 48.7 |
| Glucose metabolism (Fasting glucose) | mmol/L | 19 | −0.08 ± 0.34 | **18** | −0.09 ± 0.33 |
| Glucose metabolism (Insulin) | µu/ml | 19 | −0.64 ± 0.67 | **18** | −1.72 ± 0.93 |
| Glucose metabolism (HOMA-IR)- homeostatic model of assessment for insulin resistance |  | 19 | −0.17 ± 0.17 | **18** | −0.57 ± 0.22 |

State details if outcomes were only described in text or figures.

***Section 5. Other information***

|  | **Description as stated in paper** |
| --- | --- |
| **Key conclusions of study authors** | In conclusion, six weeks of daily supplementation with a combined formulation, comprising extracts  of olive leaf, green coffee bean and beetroot, did not lower 24-h ABP nor clinic BP nor improve blood lipids, blood glucose nor insulin sensitivity in adults with borderline or mildly-elevated BP |
| **Results that you calculated using a formula** | Convert lipid profile unit from mmol/l to mg/dl |
| **References to other relevant studies**  *(Did this report include any references to unpublished data from potentially eligible trials not already identified for this review? If yes, give list contact name and details)* |  |
| **Correspondence required for further study information** *(from whom, what and when)* |  |

**Sources:**

Higgins JPT, Green S (editors). Cochrane Handbook for Systematic Reviews of Interventions Version 5.1.0 [updated March 2011]. The Cochrane Collaboration, 2011.Available from www.cochrane-handbook.org.
