# Supplementary material for: Olive leaf extract effect on cardiometabolic profile among adults with prehypertension and hypertension: a systematic review and meta-analysis
Source: PeerJ. 2021 Apr 7;9:e11173. doi: 10.7717/peerj.11173 (PMC8035902; doi:10.7717/peerj.11173)
Supplement: Supplemental Information 5 [file peerj-09-11173-s005.docx]

***Study Eligibility & Data Collection Form***

***General Information***

| **Study ID**  *(e.g. author name, year)* | Endang Susalit, 2011 |
| --- | --- |
| **Form completed by** | Muhammad Asyraf Bin Ismail |
| **Study author contact details** | asyraf88fm@gmail.com |
| **Publication type**  *(e.g. full report, abstract, letter)* | Full Report |
| **List of included publications** |  |
| **References of similar trial*** |  |

*This is when the authors published the same study in several reports. All these references to a similar trial should be linked under one *Study ID* in RevMan.

***Study eligibility***

|  | Yes | No | Unclear | Further details |
| --- | --- | --- | --- | --- |
| **RCT/Quasi/CCT** | ***/*** |  |  |  |
| **Relevant participants** | ***/*** |  |  |  |
| **Relevant interventions** | ***/*** |  |  |  |
| **Relevant outcomes*** | ***/*** |  |  |  |

*Include only if the presence of outcomes form the inclusion criterion

If the above answers are ‘YES’, proceed to Section 1.

If any of the above answers are ‘NO*’, record below the information for ‘Excluded studies’

| Reason(s) for exclusion |
| --- |
|  |

Section 1. Characteristics of included studies

This section is to be completed by only one reviewer. State initials: ……

| **METHODS** | **Descriptions as stated in paper** |
| --- | --- |
| **Aim of study** *(e.g. efficacy, equivalence, pragmatic)* | To evaluate the anti-hypertensive effect as well as the tolerability of Olive leaf extract in comparison with Captopril in patients with stage-1 hypertension. Additionally, this study also investigated the hypolipidemic effects of Olive leaf extract in such patients |
| **Design** *(e.g. parallel, crossover, cluster)* | Randomized, double-blind, active-controlled clinical  study |
| **Unit of allocation**  *(by individuals, cluster/ groups or body parts)* | Individuals |
| **Start & end dates** | October 2007 to August 2008 |
| **Total study duration** | 12 weeks (4-week single-blind placebo (is diet-alone) run-in period and followed by a 8-week double-blind treatment period with: active control drug (Captopril) or Olive leaf extract) |
| **Sources of funding**  *(including role of funders)* | PT Dexa Medica and Frutarom Switzerland Ltd |
| **Possible conflicts of interest**  *(for study authors)* | Not stated |

| **PARTICIPANTS** | **Description**  *(include information for each intervention or comparison group)* |
| --- | --- |
| **Population description**  *(Company/companies; occupation)* | Stage 1 hypertension |
| **Setting**  *(including location (city, state, country) and single centre / multicenter)* | Jakarta Indonesia  Single center- Nephrology & Hypertension Division, Department of Internal Medicine, of Medicine, University of Indonesia |
| **Inclusion criteria** | stage-1 hypertension, as defined by clinic SBP of140–159mmHg, with DBP of either <90mmHg (who were classified as isolated systolic hypertension, ISH) or in between 90 and 99mmHg, at screening and after the run-in period visit, either naïve or being under treatment with any anti-hypertensive medication, aged between 25 and 60 years old at screening. |
| **Exclusion criteria** | history of secondary hypertension, such as hyperaldosteronism, pheochromocytoma, renal artery stenosis, cushing syndrome; presence of target-organ damage (renal failure, congestive heart failure, myocardial infarction or cerebrovascular accident 6 months preceeding to the study), second- or third-degree heart block, valvular heart disease; diabetic subjects; hepatic dysfunction; any disease state which judged by the investigator could interfere with trial participation or trial evaluation; known or suspected allergy to the trial product or the related products; and participation in any other clinical studies within 30 days prior to screening. Pregnant and breast-feeding female subjects were not allowed to participate. |
| **Method of recruitment of participants** *(e.g. phone, mail, clinic patients, voluntary)* | Clinic- Nephrology & Hypertension Division, Department of Internal Medicine, of Medicine, University of Indonesia |
| **Total no. randomised** | 179 |
| **Clusters**  *(if applicable, no., type, no. people per cluster)* | None |
| **No. randomised per group**  *(specify whether no. people or clusters)* | Intervention: 90  Control: 89 |
| **No. missing**  *(if overall, e.g. exclusions & withdrawals, whether or not missing from analysis)* | Intervention: 18  Control: 13 |
| **Reasons missing** | Intervention: Eight were due to non-compliance, six were due to ineffective therapy, one was due to an adverse effect, and three were due to other reasons  Control: Eight were due to non-compliance, two were due to ineffective therapy, one was a screened failure, and two were due to other reasons |
| **Baseline imbalances** |  |
| **Age** | OLE: 51.5 (5.8)  Captopril: 49.7 (6.8) |
| **Sex (proportion)** | OLE: Male- 12 Female- 60  Captopril: Male-10 Female-66 |
| **Race/Ethnicity** | Not stated |
| **Other relevant sociodemographics** | None |
| **Subgroups measured** *(eg split by age or sex)* | None |
| **Subgroups reported** | None |

Section 2. Risk of bias assessment

We recommend you refer to and use the method described in the Cochrane Handbook.

This section is completed by two reviewers. State initials: (i)…… (ii) ……

| **Domain** | **Risk of bias** | **Support for judgement**  *(include direct quotes where available with explanatory comments)* | **Location in text or source** *(page, table)* |
| --- | --- | --- | --- |
|  | Low/High/Unclear |  |  |
| **Random sequence generation**  *(selection bias)* | Low | Eligible patients were enrolled in the study and instructed to follow a dietary advice during their participation in the study. After a 4week run period, those who still eligible according to the inclusion and exclusion criteria would receive a randomization number allocating them to receive either active control or study drugs. | Page 252 |
| **Allocation concealment**  *(selection bias)* | Low | Randomization number allocating them to active control or study group. Further method not explained. | Page 252 |
| **Blinding of participants and personnel**  *(performance bias)* | Low | Study medications were given in a double-blind double dummy  fashion. Dummies of each medication contained the same ingredients as the respective active preparations but without the active substances.  after 2 weeks in 8 weeks of intervention, they double the dose of comparator (captopril) in patient who did not show a BP reduction in which unlikely the health personnel were properly blinded. | Page 253 |
| **Blinding of outcome assessment**  *(detection bias)* | Low | Objective outcome measurement unlikely to be influenced |  |
| **Incomplete outcome data**  *(attrition bias)* | Low | The missing data between group were balanced and due to incompliance, 6 in OLE and 8 in captopril. The missing data were properly shown in flow chart | Page 254 |
| **Selective outcome reporting**  *(reporting bias)* | Low | All the outcomes are reported |  |
| **Other bias** | Low |  | Page 253 |

Random sequence generation = Process used to assign people into intervention and control groups

Allocation concealment = Process used to prevent foreknowledge of group assignment in a RCT

Blinding of participants and personnel = Presence or absence of blinding for participants and health personnel

Blinding of outcome assessment = presence or absence of blinding for assessment of outcome

Incomplete outcome data = application of intention-to-treat analysis is one in which all the participants in a trial are analysed according to the intervention to which they were allocated

Selective outcome reporting = Selection of a subset of the original variables recorded

***Section 3. Intervention groups***

This section is completed by two reviewers. State initials: (i)…… (ii) NMN

| **Outcomes relevant to your review**  *(Copy and paste from ‘Types of outcome measures’)* | **Reported in paper**  *(Yes / No)* | **Outcome definition** *(with diagnostic criteria if relevant)* | **Unit of measurement & tool**  *(if relevant)* | **Reanalysis required?** *(specify)* |
| --- | --- | --- | --- | --- |
| Systolic blood pressure | Yes | Changes in clinical SBP | mmHg |  |
| Diastolic blood pressure | Yes | Changes in clinical DBP | mmHg |  |
| Lipid profile | Yes | 1) Total cholesterol  2) LDL  3) HDL  4) TG | mg/dl  mg/dl  mg/dl  mg/dl |  |
| Inflammatory markers for CVD | No | 1) IL-6  2) IL-8  3) TNF-alpha | ng/L  ng/L  ng/L |  |
| Glucose metabolism | No | 1) Fasting glucose  2) Insulin  3) HOMA-IR (insulin resistance) | mmol/L  µu/ml  no unit |  |
| Safety (Creatinine, AST, ALT) | Yes | 1) Creatinine  2) AST  3) ALT | mg/dl  U/L  U/L |  |
| Outcome 7 |  |  |  |  |
| Outcome 8 |  |  |  |  |

***Section 4. Data and analysis***

| **DICHOTOMOUS OUTCOME** | Intervention group | | Control group | |
| --- | --- | --- | --- | --- |
|  | Number of events | Number of participants | Number of events | Number of participants |
|  |  |  |  |  |
|  |  |  |  |  |
|  |  |  |  |  |
|  |  |  |  |  |
|  |  |  |  |  |
|  |  |  |  |  |

State details if outcomes were only described in text or figures.

| **CONTINUOUS OUTCOME** | Unit of measurement | Intervention group | | Control group | |
| --- | --- | --- | --- | --- | --- |
|  |  | n | Mean (SD) | n | Mean (SD) |
| Systolic blood pressure | mmHg | 72 | −11.5 (8.6) | **76** | −13.7 (7.7) |
| Diastolic blood pressure | mmHg | 72 | −4.8 (5.5) | **76** | −6.4 (5.2) |
| Lipid profile (TC) | mg/dl | 72 | −5.8 (22.2) | **76** | 0.5 (17.4) |
| Lipid profile (LDL) | mg/dl | 72 | −3.9 (19.4) | **76** | 2.1 (14.2) |
| Lipid profile (HDL) | mg/dl | 72 | 0.1 (5.7) | **76** | −0.9 (5.4) |
| Lipid profile (TG) | mg/dl | 72 | −11.9 (46.2) | **76** | −1.3 (43.3) |
| Safety (creatinine) | mg/dl | 72 | Cr at baseline: 0.81 (0.68)  Cr at end of treatment: 0.76 (0.16) | **76** | Cr at baseline: 0.70 (0.16)  Cr at end of treatment: 0.74 (0.16) |
| Safety (ALT)-liver function | U/L | 72 | ALT level at baseline: 20.0 (11.0)  ALT level at end of treatment: 18.2 (9.4) | **76** | ALT level at baseline: 18.5 (9.6)  ALT level at end of treatment: 17.1 (9.5) |
| Safety (AST)-liver function | U/L | 72 | AST level at baseline: 21.3 (5.9)  AST level at end of treatment: 20.5 (5.5) | **76** | AST level at baseline: 19.4 (5.8)  AST level at end of treatment: 18.6 (5.0) |

State details if outcomes were only described in text or figures.

***Section 5. Other information***

|  | **Description as stated in paper** |
| --- | --- |
| **Key conclusions of study authors** | Olive (Olea europaea L.) leaf extract at the dosage regimen of 500mg twice daily (1000mg daily) effectively lowered systolic and diastolic blood pressures in subjects with stage-1 hypertension. The anti-hypertensive activity of the extract was comparable to that of Captopril, given at its effective dose of 12.5–25mg twice daily. The study also demonstrated the safety and tolerability of the extract. Additionally, the beneficial effects of the extract on lipid profile, particularly in reducing plasma LDL-, total-cholesterol and triglyceride levels were strongly indicated by this trial. |
| **Results that you calculated using a formula** | None |
| **References to other relevant studies**  *(Did this report include any references to unpublished data from potentially eligible trials not already identified for this review? If yes, give list contact name and details)* | None |
| **Correspondence required for further study information** *(from whom, what and when)* | - |

**Sources:**

Higgins JPT, Green S (editors). Cochrane Handbook for Systematic Reviews of Interventions Version 5.1.0 [updated March 2011]. The Cochrane Collaboration, 2011.Available from www.cochrane-handbook.org.
