# Supplementary material for: Olive leaf extract effect on cardiometabolic profile among adults with prehypertension and hypertension: a systematic review and meta-analysis
Source: PeerJ. 2021 Apr 7;9:e11173. doi: 10.7717/peerj.11173 (PMC8035902; doi:10.7717/peerj.11173)
Supplement: Supplemental Information 6 [file peerj-09-11173-s006.docx]

***Study Eligibility & Data Collection Form***

***General Information***

| **Study ID**  *(e.g. author name, year)* | Tania Perrinjaquet-Moccetti, 2008 |
| --- | --- |
| **Form completed by** | Muhammad Asyraf Bin Ismail |
| **Study author contact details** | asyraf88fm@gmail.com |
| **Publication type**  *(e.g. full report, abstract, letter)* | Full Report |
| **List of included publications** |  |
| **References of similar trial*** |  |

*This is when the authors published the same study in several reports. All these references to a similar trial should be linked under one *Study ID* in RevMan.

***Study eligibility***

|  | Yes | No | Unclear | Further details |
| --- | --- | --- | --- | --- |
| **RCT/Quasi/CCT** | ***/*** |  |  |  |
| **Relevant participants** | ***/*** |  |  |  |
| **Relevant interventions** | ***/*** |  |  |  |
| **Relevant outcomes*** | ***/*** |  |  |  |

*Include only if the presence of outcomes form the inclusion criterion

If the above answers are ‘YES’, proceed to Section 1.

If any of the above answers are ‘NO*’, record below the information for ‘Excluded studies’

| Reason(s) for exclusion |
| --- |
|  |

Section 1. Characteristics of included studies

This section is to be completed by only one reviewer. State initials: ……

| **METHODS** | **Descriptions as stated in paper** |
| --- | --- |
| **Aim of study** *(e.g. efficacy, equivalence, pragmatic)* | Food Supplementation with an Olive (Olea europaea L.) Leaf Extract Reduces Blood Pressure in Borderline Hypertensive Monozygotic Twins |
| **Design** *(e.g. parallel, crossover, cluster)* | open, controlled, parallel-group, co-twin study |
| **Unit of allocation**  *(by individuals, cluster/ groups or body parts)* | Groups |
| **Start & end dates** | Not stated |
| **Total study duration** | 8 weeks |
| **Sources of funding**  *(including role of funders)* | Frutarom Switzerland Ltd |
| **Possible conflicts of interest**  *(for study authors)* | No |

| **PARTICIPANTS** | **Description**  *(include information for each intervention or comparison group)* |
| --- | --- |
| **Population description**  *(Company/companies; occupation)* | Borderline hypertensive patient |
| **Setting**  *(including location (city, state, country) and single centre / multicenter)* | Berlin Germany  Single center- HealthTwisSt GmbH |
| **Inclusion criteria** | Untreated suboptimal blood pressure, exceeding 120 mmHg systolic or 80 mmHg diastolic at rest |
| **Exclusion criteria** | Not stated |
| **Method of recruitment of participants** *(e.g. phone, mail, clinic patients, voluntary)* | Recruited patients |
| **Total no. randomised** | 20 |
| **Clusters**  *(if applicable, no., type, no. people per cluster)* | None |
| **No. randomised per group**  *(specify whether no. people or clusters)* | Intervention: 10  Control: 10 |
| **No. missing**  *(if overall, e.g. exclusions & withdrawals, whether or not missing from analysis)* | Intervention: none  Control: none |
| **Reasons missing** | none |
| **Baseline imbalances** |  |
| **Age** | OLE: 35.7 ± 14.8  Control (lifestyle modification): 38.1 ± 14.7 |
| **Sex (proportion)** | OLE: not stated  control: Male-3 Female-7 |
| **Race/Ethnicity** | Not stated |
| **Other relevant sociodemographics** | None |
| **Subgroups measured** *(eg split by age or sex)* | None |
| **Subgroups reported** |  |

Section 2. Risk of bias assessment

We recommend you refer to and use the method described in the Cochrane Handbook.

This section is completed by two reviewers. State initials: (i)…… (ii) ……

| **Domain** | **Risk of bias** | **Support for judgement**  *(include direct quotes where available with explanatory comments)* | **Location in text or source** *(page, table)* |
| --- | --- | --- | --- |
|  | Low/High/Unclear |  |  |
| **Random sequence generation**  *(selection bias)* | Unclear | Twin of each pair randomly assigned to different treatment group. Unclear method | Page 1239 |
| **Allocation concealment**  *(selection bias)* | Unclear | Twin of each pair randomly assigned to different treatment group. Unclear method | Page 1239 |
| **Blinding of participants and personnel**  *(performance bias)* | Unclear | Comments:…  Quotes “In experiment 1, control group received no treatment (lifestyle modification) compared to active group which received medications.” | Page 1239 to 1240 |
| **Blinding of outcome assessment**  *(detection bias)* | Low | Objective outcomes unlikely to be influenced |  |
| **Incomplete outcome data**  *(attrition bias)* | Low | Data complete |  |
| **Selective outcome reporting**  *(reporting bias)* | Low | All outcomes were reported |  |
| **Other bias** | Low |  |  |

Random sequence generation = Process used to assign people into intervention and control groups

Allocation concealment = Process used to prevent foreknowledge of group assignment in a RCT

Blinding of participants and personnel = Presence or absence of blinding for participants and health personnel

Blinding of outcome assessment = presence or absence of blinding for assessment of outcome

Incomplete outcome data = application of intention-to-treat analysis is one in which all the participants in a trial are analysed according to the intervention to which they were allocated

Selective outcome reporting = Selection of a subset of the original variables recorded

***Section 3. Intervention groups***

This section is completed by two reviewers. State initials: (i)…… (ii) NMN

| **Outcomes relevant to your review**  *(Copy and paste from ‘Types of outcome measures’)* | **Reported in paper**  *(Yes / No)* | **Outcome definition** *(with diagnostic criteria if relevant)* | **Unit of measurement & tool**  *(if relevant)* | **Reanalysis required?** *(specify)* |
| --- | --- | --- | --- | --- |
| Systolic blood pressure | Yes | Changes in clinical SBP | mmHg |  |
| Diastolic blood pressure | Yes | Changes in clinical DBP | mmHg |  |
| Lipid profile | Yes | 1) Total cholesterol  2) LDL  3) HDL  4) TG | mg/dl  mg/dl  mg/dl  mg/dl |  |
| Inflammatory markers for CVD | No | 1) IL-6  2) IL-8  3) TNF-alpha | ng/L  ng/L  ng/L |  |
| Glucose metabolism | No | 1) Fasting glucose  2) Insulin  3) HOMA-IR (insulin resistance) | mmol/L  µu/ml  no unit |  |
| Safety (Creatinine, AST, ALT) | No | 1) Creatinine  2) AST  3) ALT | mg/dl  U/L  U/L |  |
| Outcome 7 |  |  |  |  |
| Outcome 8 |  |  |  |  |

***Section 4. Data and analysis***

| **DICHOTOMOUS OUTCOME** | Intervention group | | Control group | |
| --- | --- | --- | --- | --- |
|  | Number of events | Number of participants | Number of events | Number of participants |
|  |  |  |  |  |
|  |  |  |  |  |
|  |  |  |  |  |
|  |  |  |  |  |
|  |  |  |  |  |
|  |  |  |  |  |

State details if outcomes were only described in text or figures.

| **CONTINUOUS OUTCOME** | Unit of measurement | Intervention group | | Control group | |
| --- | --- | --- | --- | --- | --- |
|  |  | n | Mean (SD) | n | Mean (SD) |
| Systolic blood pressure | mmHg | 10 | Week 0: 135 ± 8  Week 8: 130 ± 11 | **10** | Week 0: 133 ± 5  Week 8: 135 ± 11 |
| Diastolic blood pressure | mmHg | 10 | Week 0: 76 ± 6  Week 8: 77 ± 7 | **10** | Week 0: 77 ± 6  Week 8: 80 ± 7 |
| Lipid profile (TC) | mg/dl | 10 | Week 0: 4.8 ± 1.0  Week 8: 4.1 ± 1.0 | **10** | Week 0: 4.8 ± 1.3  Week 8: 3.9 ± 0.7 |
| Lipid profile (LDL) | mg/dl | 10 | Week 0: 2.8 ± 0.6  Week 8: 2.3 ± 0.7 | **10** | Week 0: 2.6 ± 0.6  Week 8: 2.0 ± 0.4 |
| Lipid profile (HDL) | mg/dl | 10 | Week 0: 1.3 ± 0.4  Week 8: 1.2 ± 0.4 | **10** | Week 0: 1.4 ± 0.4  Week 8: 1.3 ± 0.4 |

State details if outcomes were only described in text or figures.

***Section 5. Other information***

|  | **Description as stated in paper** |
| --- | --- |
| **Key conclusions of study authors** | In conclusion, the study confirmed the antihypertensive and cholesterol-lowering actions of the olive leaf extract EFLA®  943 in humans. For the 1000 mg dose these actions were substantial in subjects with borderline hypertension whereas, for the 500 mg dose, they were only detectable using the co-twin approach |
| **Results that you calculated using a formula** | Convert lipid profile unit from mmol/l to mg/dl |
| **References to other relevant studies**  *(Did this report include any references to unpublished data from potentially eligible trials not already identified for this review? If yes, give list contact name and details)* | None |
| **Correspondence required for further study information** *(from whom, what and when)* | - |

**Sources:**

Higgins JPT, Green S (editors). Cochrane Handbook for Systematic Reviews of Interventions Version 5.1.0 [updated March 2011]. The Cochrane Collaboration, 2011.Available from www.cochrane-handbook.org.
