# Supplementary material for: Olive leaf extract effect on cardiometabolic profile among adults with prehypertension and hypertension: a systematic review and meta-analysis
Source: PeerJ. 2021 Apr 7;9:e11173. doi: 10.7717/peerj.11173 (PMC8035902; doi:10.7717/peerj.11173)
Supplement: Supplemental Information 7 [file peerj-09-11173-s007.docx]

**Systematic Review and/or Meta-Analysis Rationale**

1. The rationale for conducting the systematic review / meta-analysis:

Olive leaf is said to have a beneficial effect on cardiometabolic profile and was used in treatment before the modern age of medicine. It can act as a natural supplement in patient management especially among patient with elevated blood pressure (prehypertension and hypertensive group) as lifestyle modifications are advocated in both groups. Thus, the aim of our review is to assess the effectiveness of olive leaf extract as a supplement on cardiometabolic profile among prehypertension and hypertensive groups.

2. The contribution that it makes to knowledge in light of previously published related reports, including other meta-analyses and systematic reviews:

A published nutrition review, stated the potential benefit of bioactive compounds in olive leaf extract which are antioxidant, antihypertensive, antiatherogenic, anti-inflammatory, hypoglycemic, and hypocholesterolemic (El & Karakaya 2009). This was further supported by another literature review which also stated the similar beneficial effects of olive leaf on human health (Vogel et al. 2015). This is because of the presence of polyphenols which are the bioactive compounds of olive leaf. At level of animal trials, a systematic review and meta- analysis of olive leaf extract on glucose levels in diabetes induced rats showed improvement in both the lipid profile and glycemic control (Abunab et al. 2017). Therefore, it shows that olive leaf extract has a lot of favorable outcomes on human health.

**References**

Abunab H, Dator WL, and Hawamdeh S. 2017. Effect of olive leaf extract on glucose levels in diabetes-induced rats: A systematic review and meta-analysis. *J Diabetes* 9:947-957. 10.1111/1753-0407.12508

El SN, and Karakaya S. 2009. Olive tree (Olea europaea) leaves: potential beneficial effects on human health. *Nutrition Reviews* 67:632-638. 10.1111/j.1753-4887.2009.00248.x

Vogel P, Machado IK, Garavaglia J, Zani VT, de Souza D, and Dal Bosco SM. 2015. Polyphenols benefits of olive leaf (Olea europaea L) to human health. *Nutr Hosp* 31:1427-1433.
